# Metabolism‐related lncRNAs signature to predict the prognosis of colon adenocarcinoma

**DOI:** 10.1002/cam4.5412

**Published:** 2022-11-10

**Authors:** Yimin Sun, Bingyan Liu, BaoLai Xiao, XueFeng Jiang, Jin‐Jian Xiang, Jianping Xie, Xiao‐Miao Hu

**Affiliations:** ^1^ Surgery Department of Gastrointestinal The First Affiliated Hospital of Yangtze University Jingzhou Hubei People's Republic of China

**Keywords:** colon adenocarcinoma, lncRNA, metabolism, prognosis

## Abstract

**Background:**

Cell metabolism and long noncoding RNA (lncRNA) played crucial roles in cancer development. However, their association in colon adenocarcinoma (COAD) remains unclear.

**Methods:**

The COAD gene expression data and corresponding clinical data were retrieved from The Cancer Genome Atlas (TCGA) database. Differential expression of metabolic genes and lncRNA were identified by comparing tumor and normal colon tissues. Pearson correlation analysis was performed to identify metabolism‐associated lncRNA. COAD patients were divided into training cohort and validation cohort by randomization. Then, a univariate Cox regression analysis was introduced to evaluate the correlations between metabolism‐related lncRNAs and overall survival (OS) of the patients in the training cohort. The least absolute shrinkage and selection operator (LASSO) method was introduced to determine and establish a prognostic prediction model. Subsequently, survival analysis, receiver operating characteristic (ROC) curve analysis, and Cox regression analysis were generated to estimate the prognostic role of the LncRNA risk score in training, validation, and entire cohorts.

**Results:**

We identified 152 differentially expressed metabolism‐associated lncRNAs (MRLncRNAs). A prognostic prediction model involving four metabolism‐related lncRNAs were established using LASSO. In each cohort, COAD patients in the high‐risk group had worse OS compared to those in the low‐risk group. The ROC analyses demonstrated that the lncRNA signature performed well in predicting OS. Uni‐ and multivariate analysis indicated that the lncRNA signature as an independent prognostic factor. Furthermore, a correlation analysis demonstrated that *LINC01138* was the most closely lncRNA related to metabolic genes. In vitro assays demonstrated that *LINC01138* affects tumor progression in COAD.

**Conclusions:**

In summary, we established a metabolism‐associated lncRNAs model to predict the prognosis in COAD patients.

## 1 INTRODUCTION

Colon adenocarcinoma (COAD) is the most prevalent histopathological type of colorectal cancer with more than a million newly diagnosed cases in 2018.^1^ With the advancement of early screening and treatment strategies, the survival of COAD patients has improved.^2^ However, for some patients with advanced tumors, the prognosis is still poor.^3^ Additionally, patients with similar clinical characteristics have significant differences in survival outcomes and quality of life.^4^ Some risk biomarkers, including CA199 and CEA have been shown to be associated with COAD, but it is not suitable for all patients.^5^ Presently, TNM staging, a tool based on clinical features is widely used to evaluate the prognosis of patients in clinical, but it is limited to reveal the biological heterogeneity of tumors.^5^ Therefore, it is meaningful to explore reliable and convenient indicators of prediction in COAD.

Cancer cells exhibit a distinct metabolism profile compared with normal cells, which is an important hallmark of cancer. In the last few decades, it had become evident that alteration of metabolism in cancer cells contributes to oncogenesis and cancer progression.^6^ It has been established that cancer cells prefer to perform glycolysis even under sufficient oxygen tension, a phenomenon known as Warburg effect or aerobic glycolysis, which supports the growth and proliferation of cancer cells.^7^ Altered aerobic glycolysis has been validated in many tumors, and targeting intervention of Warburg effect can inhibit tumor progression. Hexokinases 2 (HK2) is the first and rate‐limiting enzyme of glycolysis, which is responsible for the first step of glycolysis. In human glioblastoma multiforme (GBM), Amparo Wolf et al. showed that HK2 is aberrantly expressed in GBM and correlated with worse survival of patients. HK2 depletion resulted in restored oxidative glucose metabolism and increased sensitivity to apoptosis of GBM cells.^8^ In addition, several glycolysis inhibitors have been introduced to target the Warburg effect. For example, acting as an inhibitor of hexokinase, administration of 3‐BrOP decreased ATP levels, induced cell death of neuroblastoma in vitro, and reduced neuroblastoma weight in vivo.^9^ In COAD, a study reported that machilin A (MA) can impair activity of LDHA, an important enzyme involvement in aerobic glycolytic, which led to decrease lactate production and ATP levels in vitro, and suppressed tumor growth in vivo.^10^ Besides, other than the aberrant metabolism, for example, amino acids metabolism has been validated to be associated with cancer progression.^11^ Thus, clarifying the role of metabolism may improve the prediction of prognostic for COAD patients and finding new potential targets for COAD.

LncRNA is a class of noncoding RNA with more than 200 nucleotides in length.^12^ It has been proposed that lncRNAs widely involved in diverse biological processes, including cell proliferation, development, and apoptosis.^13^, ^14^, ^15^ Moreover, the dysregulation expression of lncRNA has been demonstrated to be associated with many diseases,^16^ including COAD.^17^ For instance, LncRNA *BLACAT1* by epigenetically silencing of *p15* affects colorectal cancer cells proliferation, which resulted in a poor outcome.^18^ lncRNA *SNHG15* through interacting with and maintaining slug stability to promote colon cancer progression.^19^ lncRNAs are a key factor in regulate cancer metabolism. A good example is lncRNA ANRIL, abnormally expressed in acute myeloid leukemia (AML). ANRIL by inhibiting the level of adiponectin receptor (AdipoR1), a key regulator of glucose metabolism, thus affected glucose uptake and cell maintenance in AML.^20^ However, the association between lncRNA and metabolism in COAD is unclarified.

Herein, we intended to explore the association between lncRNA and metabolism and establish a reliable metabolism‐associated lncRNAs signature for predicting the prognosis of COAD patients. Finally, we validated the role of a representative metabolism‐associated lncRNAs in COAD.

## 2 MATERIALS AND METHODS

### 2.1 Dataset processing

RNA sequencing data of 398 tumor and 39 normal colon tissues, and corresponding clinical features were obtained from TCGA database (https://www.cbioportal.org) on February 10, 2022. Due to the incomplete clinical characteristics of some patients in COAD, we reanalyzed the data and excluded COAD patients without complete clinical characteristics. Overall, a total of 364 COAD patients were included.

### 2.2 Identification of metabolism‐related lncRNAs

Cell metabolism genes were extracted from ccmGDB (http://bioinfo.mc.vanderbilt.edu/ccmGDB). LncRNA annotation was obtained from Gencode (www.gencodegenes.org/). Differentially expressed metabolic genes and lncRNAs were performed by “limma” package (|log2FoldChange| >1 and false discovery rate [FDR] < 0.05 as statistical significance). Based on the criteria of |correlation coefficient| ≥ 0.3 and *p* < 0.001 by Pearson correlation analysis, we identified metabolism‐related lncRNAs.

### 2.3 Establish the metabolism‐related lncRNAs prognostic signatures

All eligible patients were randomly divided into training cohort with 183 patients and validation cohort with 181 patients by the R package “caret”^21^ (Table S1). Table 1 displays clinical features of COAD patients in all cohorts. First, R package “survival” was introduced to characterize the significant MRlncRNAs in training cohort. Subsequently, the LASSO Cox regression analysis was introduced to refine genes by R package “glmnet.” Ultimately, four lncRNAs were selected to establish a metabolism‐associated lncRNAs model. The risk score of each sample were calculated for each subject as follows: Risk Score = βlncRNA1 × ExplncRNA1 + βlncRNA2 × ExplncRNA2 + … βlncRNAn×ExplncRNAn (β: the coefficient from LASSO Cox regression analysis, Exp: gene expression).^22^, ^23^ The median value was used as the cutoff value, and the patients were classified as low risk and high risk accordingly.

**TABLE 1:** Characteristics of COAD patients

	No. (%)			
Characteristics	Entire cohort (*n* = 364)	Training cohort (*n* = 183)	Validation cohort (*n* = 181)	*p* value
**Age, years**				0.538
≤60	110 (30.2)	58 (31.7)	52 (28.7)	
>60	254 (69.8)	125 (68.3)	129 (71.3)	
**Gender**				0.073
Female	168 (46.2)	93 (50.8)	75 (41.4)	
Male	196 (53.8)	90 (49.2)	106 (58.6)	
**AJCC stage**				0.523
I	65 (17.8)	34 (18.6)	31 (17.1)	
II	144 (39.6)	67 (36.6)	77 (42.5)	
III	99 (27.2)	51 (27.9)	48 (26.5)	
IV	51 (14.0)	27 (14.7)	24 (13.3)	
Unknown	5 (1.4)	4 (2.2)	1 (0.6)	
**Stage T**				0.829
T1	9 (2.5)	4 (2.2)	5 (2.8)	
T2	65 (17.8)	34 (18.6)	31 (17.1)	
T3	250 (68.7)	123 (67.2)	127 (70.2)	
T4	39 (10.7)	21 (11.5)	18 (9.9)	
Unknown	1 (0.3)	1 (0.5)	0 (0)	
**Stage N**				0.864
N0	218 (59.9)	109 (59.6)	109 (60.2)	
N1	84 (23.1)	42 (22.9)	42 (23.2)	
N2	62 (17.0)	32 (17.5)	30 (16.6)	
**Stage M**				0.782
M0	279 (76.7)	139 (76.0)	140 (77.3)	
M1	51 (14.0)	27 (14.7)	24 (13.3)	
Unknown	34 (9.3)	17 (9.3)	17 (9.4)	
**Alive status**				0.939
Alive	289 (79.4)	145 (79.2)	144 (79.6)	
Died	75 (20.6)	38 (20.8)	37 (20.4)	

### 2.4 Function prediction of the lncRNA signature

In general, LncRNA has no ability of coding protein, and it plays a role by regulating the expression of coding genes.^12^ We indirectly clarified the function of lncRANs by investigating the metabolic genes related to lncRANs. The R package of clusterProfiler was employed to conduct a KEGG pathway and GO enrichment analysis.

### 2.5 Cell culture

Human colon adenocarcinoma cell lines LoVo (CCL‐229), HCT116 (CCL‐247), HT‐29 (HTB‐38), and SW480 (CCL‐228) were obtained from ATCC (Rockville, MD). Cells were cultured in the McCoy's 5A, F‐12 K, and Leibovitz's L‐15 medium (containing 12% fetal bovine serum, 100 U/ml penicillin, and 100 μg/ml streptomycin) at 37°C in a 5% CO2 incubator.

### 2.6 Gene overexpression and knockdown

Human genes *LINC01138* (Gene ID: 388685) overexpression vector with pGPU6 was purchased from by GenePharma (Shanghai, China). Oligonucleotides specific for shRNAs against *LINC01138* were designed and purchased from Genechem (Shanghai, China) with pGPU6 vector. The sequences were: sh‐*LINC01138* #1: 5′‐GCACATTTGAGATACTGTTCAAGAGACAGTATCTCAAATGTGCTTTTTT‐3′ (forward) and 5′‐AAAAAAGCACATTTGAGATACTGTCTCTTGAACAGTATCTCAAATGTGC′ (reverse); sh‐*LINC01138* #2: 5′‐GATGGAGTCTCACTGTTCAAGAGACAGTGAGACTCCATCTTTTTT‐3′(forward) and 5′‐AAAAAAGATGGAGTCTCACTGTCTCTTGAACAGTGAGACTCCATC′ (reverse).

### 2.7 Quantitative real‐time PCR

TRIzol reagent (Invitrogen) was used to isolate total RNA according to the manufacturer's suggestion. Then, obtained RNA (~2.0 μg) was used to synthesize cDNA with a kit (RR047A, Takara, Japan). RT‐qPCR analyses were performed on an Applied Biosystems 7500 Sequence Detector with luminaris color hiGreen qPCR master mix (Thermo Fisher Scientific). The procedures were used for the qPCR as follows: predenaturation of 95°C for 10 min, 36 cycles of 95°C for 10 s, and 60°C for 60 s. The transcript levels were normalized to β‐actin and analyzed by 2^−ΔΔCt^ method. The primers were shown below: for *LINC01138*: 5′‐TACGAAAGCTGAAAGCGTGC‐3′ (sense) and 5′‐ATGAAACAGAACTGCAAACAGGC‐3′ (antisense).

### 2.8 Western blotting

Protein extraction and was determination based on the manufacturer's instructions (C500005 and C503051, Sangon Biotech). Subsequently, the obtained proteins (~30 μg) were analyzed by SDS–PAGE and transferred onto PVDF membranes (ImmobilonP). Thereafter, the membrane was blocked for 1 h at normal temperature with nonfatty milk (3%), and the membranes were incubated at 4°C overnight with the following primary antibodies: rabbit anti‐human antibodies against DGKH (1:1000; 13,873‐1‐AP, Proteintech), PDE4D (1:3000; 67,062‐1‐Ig, Proteintech), PYCR1 (1:1000; 66,510‐1‐Ig, Proteintech), TKT (1:3000; 66,016‐1‐Ig, Proteintech), or β‐actin (1:5000; 66,009‐1‐Ig, Proteintech). Then, the membranes were washed three times at 10‐min intervals, with PBS containing 0.1% Tween‐20. Secondary antibodies were incubated for 1 h at normal temperature (1:5000, SA00001‐2, Proteintech). Finally, special bands were determined by a commercial kit (12,630, Cell Signaling Technology, Inc.). Image‐Pro Plus 6.0 imaging software (Media Cybernetics, Inc.) was used to quantify protein expression and followed statistics by GraphPad Prism 9 (GraphPad Software).

### 2.9 MTT assay

A total of 1 × 10^3^ cells (in 100 μl) was seeded into a 96‐well plate and allowed to grow for the indicated time period before analysis. Thereafter, 5 mg/mL MTT solution was added to individual wells and the 96‐well plate were incubated in a 5% CO_2_ incubator at 37°C for 4 h. Then removed the medium, 100 μl dimethylsulfoxide (DMSO) was added to dissolve the formazan crystals and the absorbance at 595 nm wavelength was measured (Bio‐Tek, USA).

### 2.10 Soft agar assay

A total of 5 × 10^3^ tumor cells were mixed with 0.4% agarose (214,230, Noble) and plated onto 6‐well plates with 0.8% solidified agar in medium containing 12% fetal bovine serum. Three weeks later, colonies underwent 100% methanol fixing and 0.5% crystal violet staining.

### 2.11 Matrigel invasion assay

The invasion ability of COAD cells was evaluated using a 24‐well Transwell plates precoated with Matrigel matrix (BD Science). Briefly, 500 μl medium (12% FBS) was added to lower chambers, and 1 × 10^5^ cells with serum‐free media was added to the top chamber. After 24 h, fixed with 4% paraformaldehyde (PFA) and was stained with 0.4% crystal violet dye. Subsequently, images from five random microscopic fields were captured, and invasive cells were examined and counted.

### 2.12 Ethical statement

This article does not contain any studies with human participants or animals performed by any of the authors.

### 2.13 Availability of data and materials

The datasets used and/or analyzed in this study are available from the corresponding author on reasonable request.

### 2.14 Statistical analysis

The differences of genes expression between COAD and normal colon tissues were investigated using the R with independent *t*‐test. R package “ggplot2” and “pheatmap” were used to plot Volcano plot and Heat maps, respectively. Survival analyzed were performed by log‐rank (Mantel–Cox) analysis. ROC curves and area under the curve (AUC) analyses were performed using “survival ROC” package. Student's test was used to compare data between two independent groups. For multiple groups comparison, one‐way analysis of variance with (ANOVA) with Tukey's correction was used. All statistics were done in GraphPad Prism 9 (GraphPad Software). *p* < 0.05 was considered statistically significant.

## 3 RESULTS

### 3.1 Identification of differentially expressed metabolism‐related lncRNAs

**FIGURE 1:**
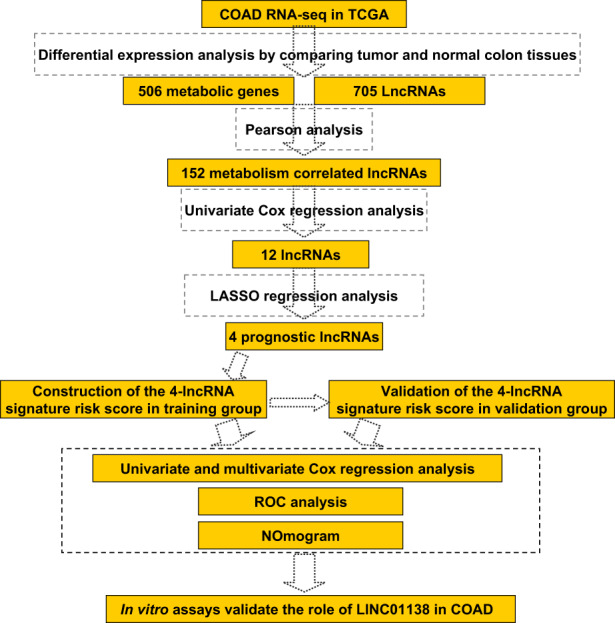
Research flowcharts.

**FIGURE 2:**
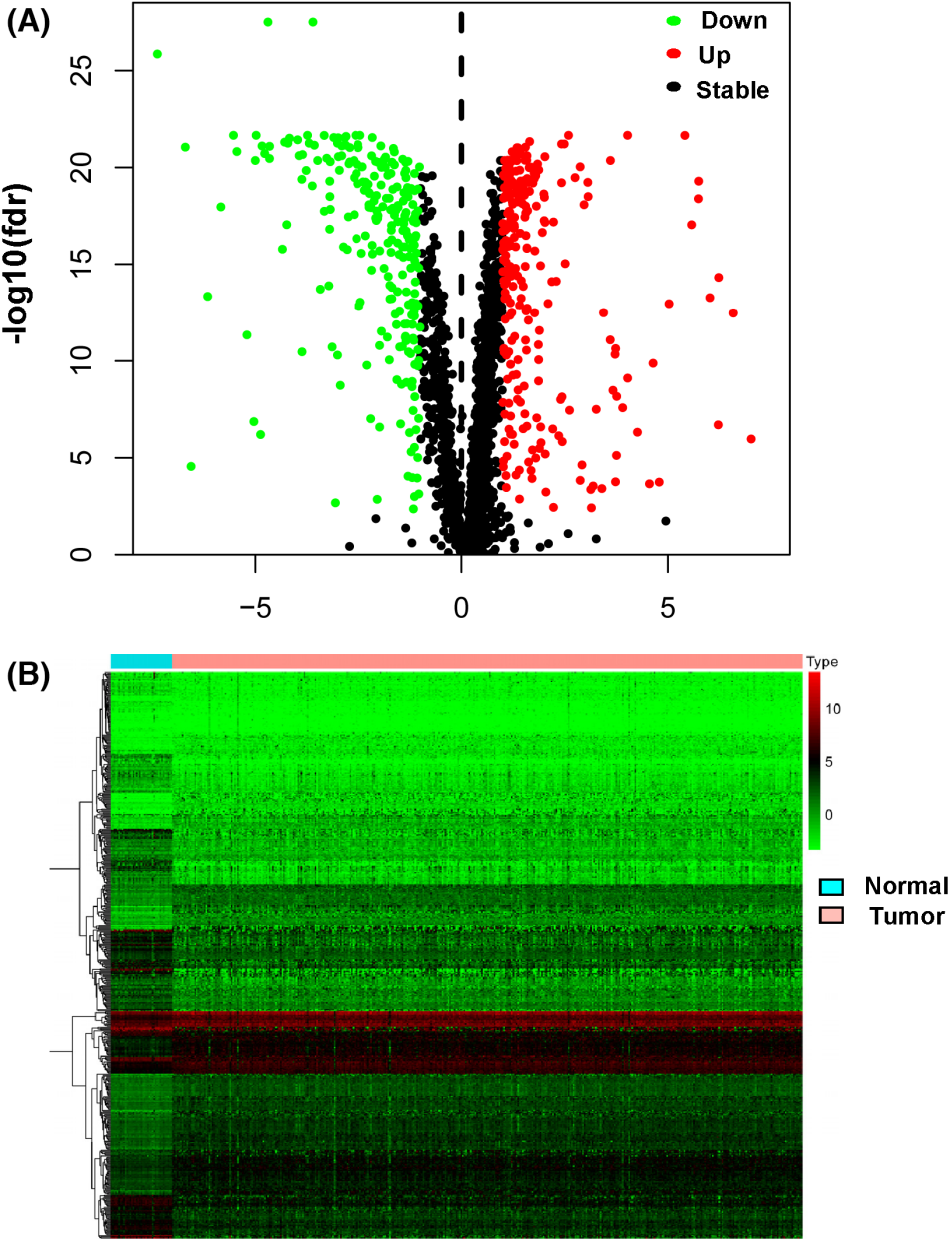
Differentially expressed metabolism‐related lncRNAs. Volcano plot (A) and heatmap (B) show 506 differentially expressed metabolism genes and 705 differentially expressed lncRNAs by comparing tumor and normal colon tissues.

Figure 1 shows the flowchart of this study. Different analyses in gene expression were performed on the TCGA COAD between 39 normal and 398 tumor tissues, and 506 differentially expressed metabolism‐related genes and 705 differentially expressed lncRNAs were identified. Subsequently, 152 metabolism‐related lncRNAs were generated by the R package “corrplot” (Figure 2A, B, and Table S2).

### 3.2 Establishment and validation of MRlncRNAs prognostic signature

**FIGURE 3:**
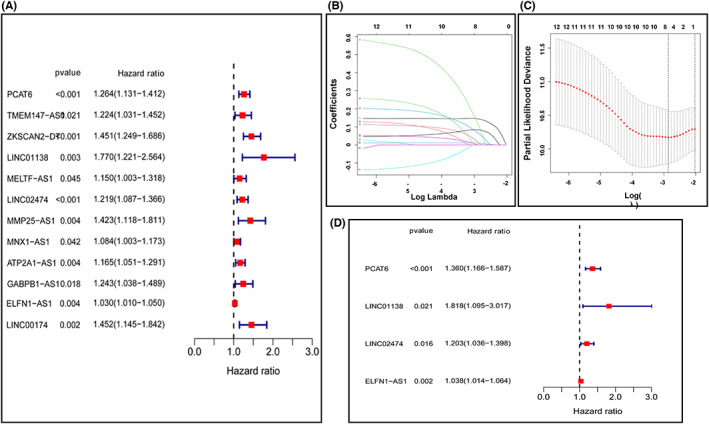
Establishment of metabolism‐related lncRNAs prognostic signature. (A) Univariate Cox regression analysis of OS for 12 metabolism‐related lncRNAs. (B and C) LASSO regression analysis was used to determine candidate metabolism‐related lncRNAs. (D) Multivariate Cox regression analysis of four metabolism‐related lncRNAs.

**FIGURE 4:**
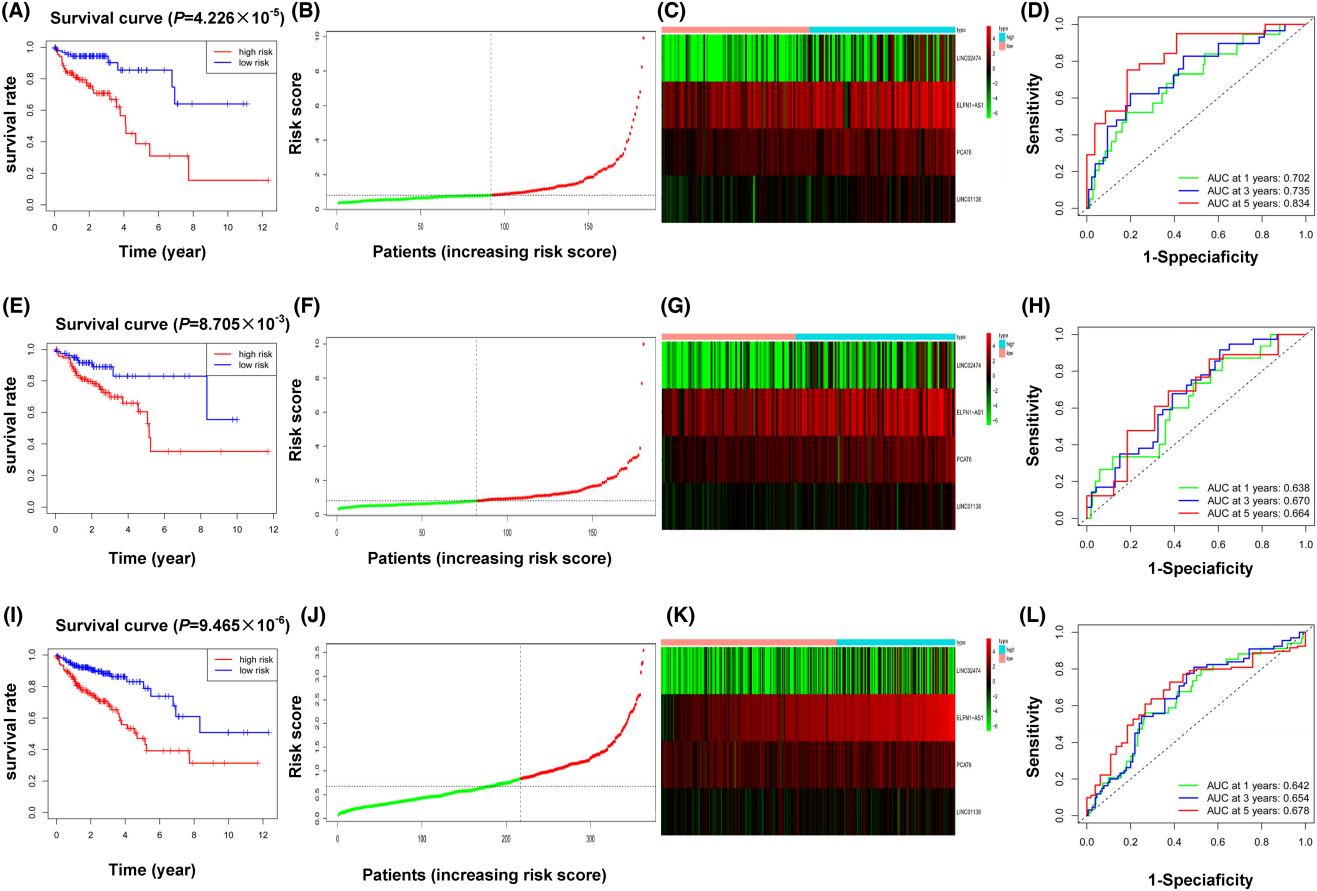
Validation of metabolism‐related lncRNAs prognostic signature. (A, E, and I) Represent the KM plot for OS of patients in different risk groups of three cohorts. (B, F, and J) Represent the risk scores distribution of gene expression pattern in three cohorts. (C, G, and K) The heatmaps show the gene expression in three cohorts. (D, H, and L) Time‐dependent ROC curves for the prognostic value of the metabolism‐related lncRNAs signature in three cohorts. Survival analysis was examined by log‐rank (Mantel–Cox) test in A, E, and I.

Univariate Cox regression analysis was introduced to indentify MRlncRNAs associated with OS in training cohort. 12 lncRNAs (*p* < 0.05) were retained, all genes (*PCAT6*, *TMEM147‐AS1*, *ZKSCAN2‐DT*, *LINC01138*, *MELTF‐AS1*, *LINC02474*, *MMP25‐AS1*, *MNX1‐AS1*, *ATP2A1‐AS1*, *GABPB1‐AS1*, *ELFN1‐AS1*, and *LINC00174)* were related with increased risk (HRs >1) (Figure 3A). To minimize the overfitting caused by univariate Cox regression, LASSO regression analysis was introduced and a 4‐gene signature was established. (Figure 3B–D). Based on the median risk score, the training cohort was classified as high‐risk group and low‐risk group accordingly. Kaplan–Meier plots demonstrated patients in the high‐risk group experienced worse OS than those in the low‐risk group (Figure 4A). The distribution patterns of risk score was shown in Figure 4B. A heatmap exhibited the expression of 4 MRlncRNAs in two groups (Figure 4C). Time‐dependent ROC curve analyses revealed that the MRlncRNAs signature has a good performance in the prediction of OS, and our result demonstrated that the AUC was 0.702, 0.735, and 0.834, for 1‐year, 3‐year, and 5‐year OS, respectively (Figure 4D). Consistent with training cohort, the validation cohort and entire cohort showed similar results, which verified the lncRNAs signature's accuracy (Figure 4E‐L).

### 3.3 Independent prognostic value of the risk score model

**FIGURE 5:**
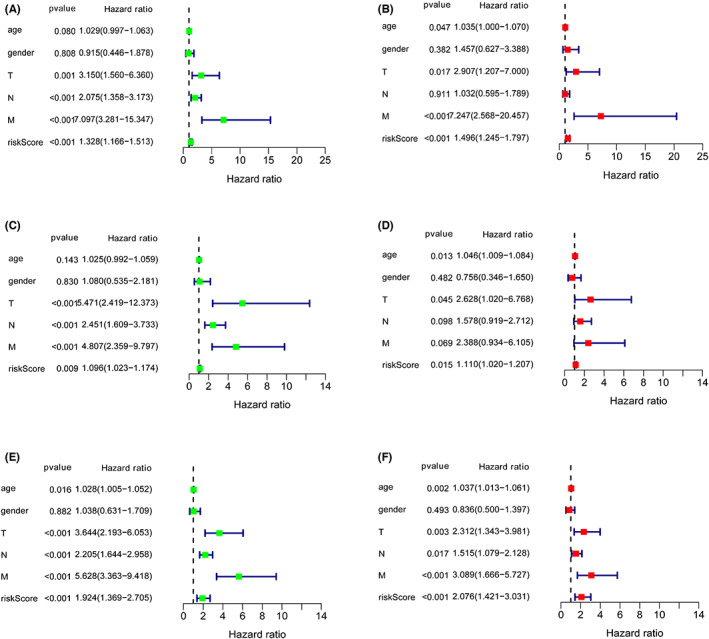
Independent prognostic value of the risk score model. (A, C, and E) Univariate Cox regression analysis of different factors in three cohorts. (B, D, and F) Multivariate Cox regression analysis of different factors in three cohorts.

To assess the prediction ability of the risk score model in COAD patients, Cox regression analyses were introduced. As shown in Figure 5A, the univariate Cox regression analyses indicated that the risk score was correlated with the OS of COAD patients, which was in line with the result of multivariate Cox regression analyses (Figure 5B). Additionally, the analyses in validation‐ and the entire‐ cohort showed similar results (Figure 5C–F).

### 3.4 Prognostic role of the MRlncRNAs signature in clinical subgroups

**FIGURE 6:**
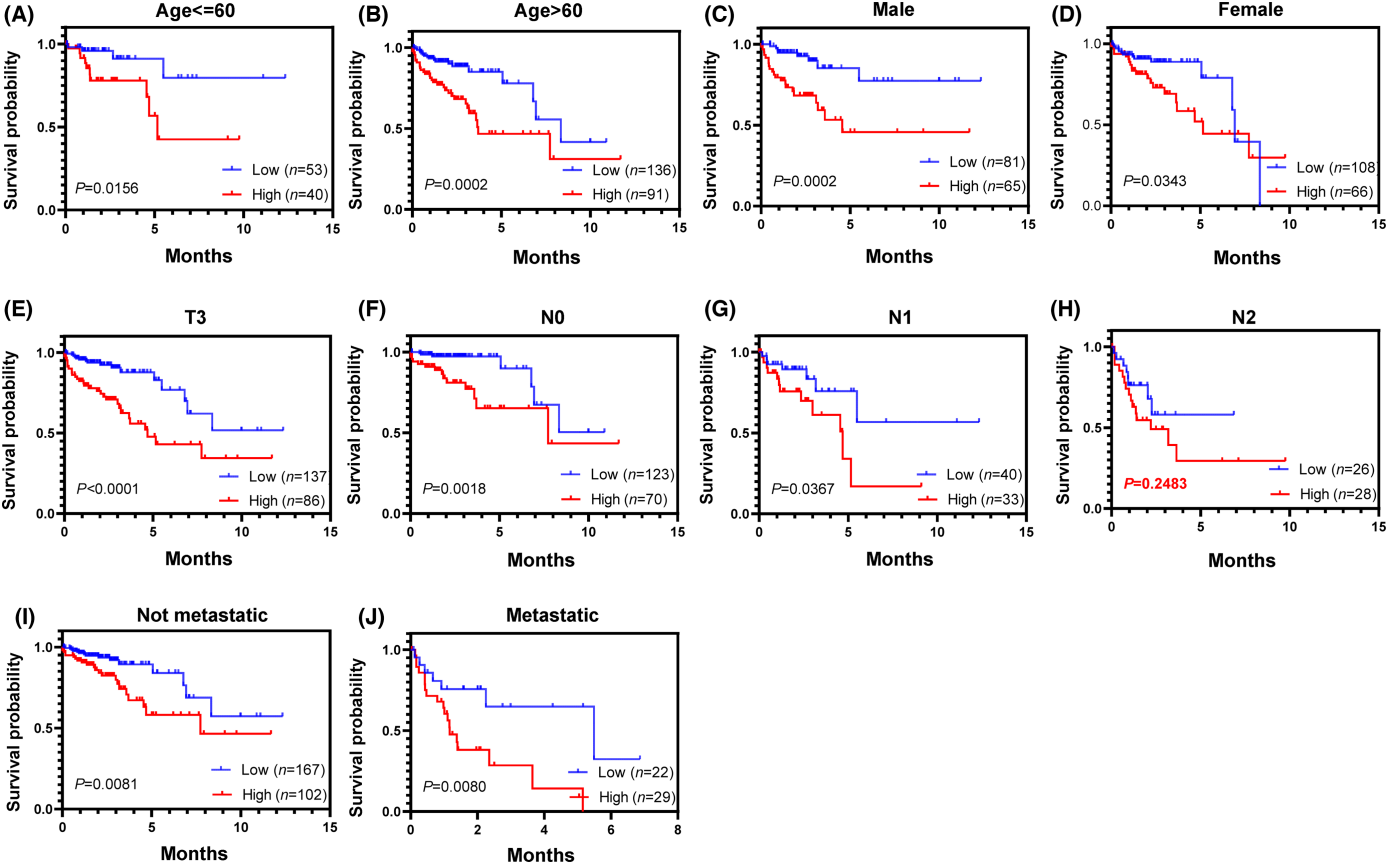
Prognostic role of the prognostic signature in clinical subgroups. (A) Age < = 60 year. (B) Age > 60 year. (C) Male. (D) Female. (E) T3. (F) N0. (G) N1. (H) N2. (I) Not metastatic. (J) Metastatic. Survival analysis was examined by log‐rank (Mantel–Cox) test in Figure 6.

To assess the prediction effciency of risk score model in clinical subgroups, stratification survival analysis was performed. The subgroups were divided according to age status (age < =60 and age > 60), gender status (female and male), tumor size status (T1, T2, T3, and T4), lymph node metastatic status (N0, N1, and N2), and distant metastatic status (not metastatic and metastatic). According to the cut‐off value of median risk score, patients were stratified into the low‐risk group and high‐risk group for OS in different subgroups, except for the N2 subgroup. Compared with the low‐risk group, patients in the high‐risk group presented worse OS (Figure 6A–J). Moreover, we did not conduct subgroup analysis for T1, T2, and T4 due to failing to divide patients into two risk groups.

### 3.5 Functional analysis of the regulatory network

**FIGURE 7:**
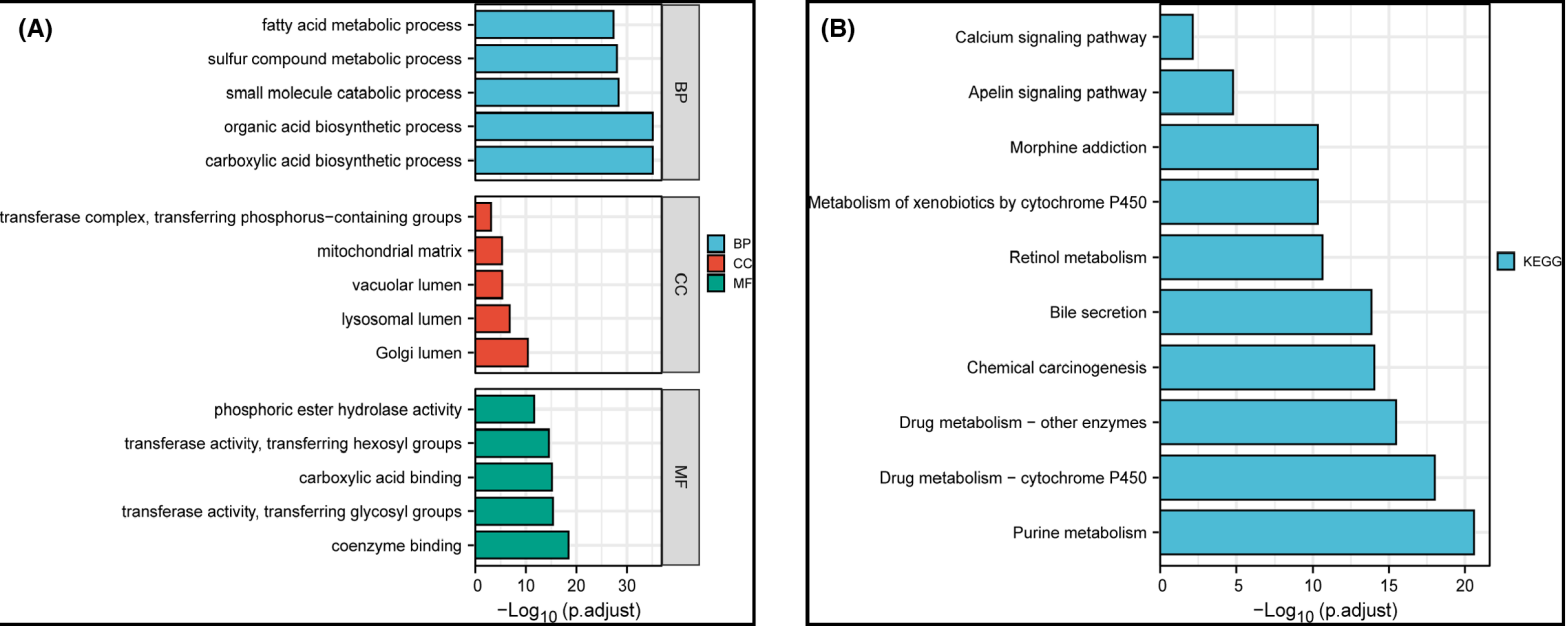
Functional enrichment analysis. (A) Gene ontology (GO) enrichment analysis of the metabolic genes related to lncRANs. CC, cellular component; MF, molecular function. (B) Kyoto Encyclopedia of Genes and Genomes (KEGG) pathway enrichment analysis of metabolic genes related to lncRANs.

As shown in Figure 7, metabolic genes related to lncRANs in the biological process (BP) category were enriched in carboxylic acid biosynthetic process, organic acid biosynthetic process, and small molecule catabolic process. In the cellular components (CC) category were mainly associated with Golgi lumen, lysosomal lumen, mitochondrial matrix, and vacuolar lumen. In the molecular functions (MF) category mainly participated in the regulation of coenzyme binding, transferase activity, transferring glycosyl groups, and carboxylic acid binding. For KEGG pathway, the main significant pathways included purine metabolism, drug metabolism, and chemical carcinogenesis, etc.

### 3.6 The expression of four MRlncRNAs in clinical subgroups

**FIGURE 8:**
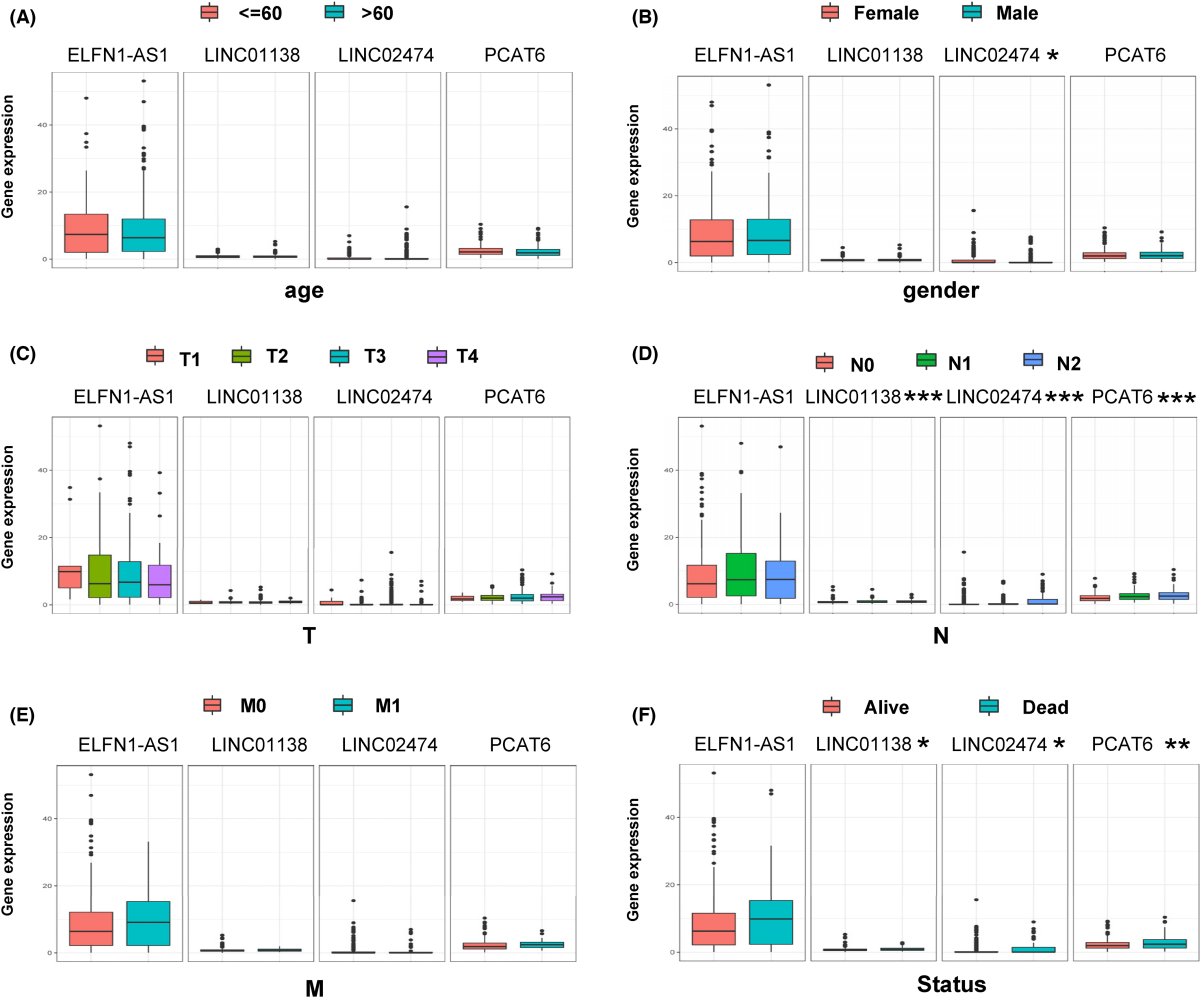
The expression of metabolism‐related lncRNAs in clinical subgroups. (A) Age. (B) Gender. (C) T: primary tumor status. (D) N: lymph node involvement status. (E) M: distant metastatic status. (F) Alive status. Unpaired two‐tailed *t*‐test was used to compare in Figure 8. *p < 0.05, **p < 0.01. ***p < 0.001.

Next, we analyzed the expression of four MRlncRNAs in different clinical subgroups. Briefly, the expression level of lncRNAs was not associated with the status of age, primary tumor size, and distant metastatic classification (Figure 8A, C, E). Only *LINC02474* showed differences in expression between the two groups in gender classification (Figure 8B). Furthermore, the expression level of *LINC01138*, *LINC02474*, and *PCAT6* showed statistical difference in expression for lymph node involvement status and alive status classification (Figure 8D, F). However, this phenomenon was not observed in *ELFN1‐AS1* (Figure 8D, F).

### 3.7 LINC01138 modulates metabolic genes in COAD cell lines

**FIGURE 9:**
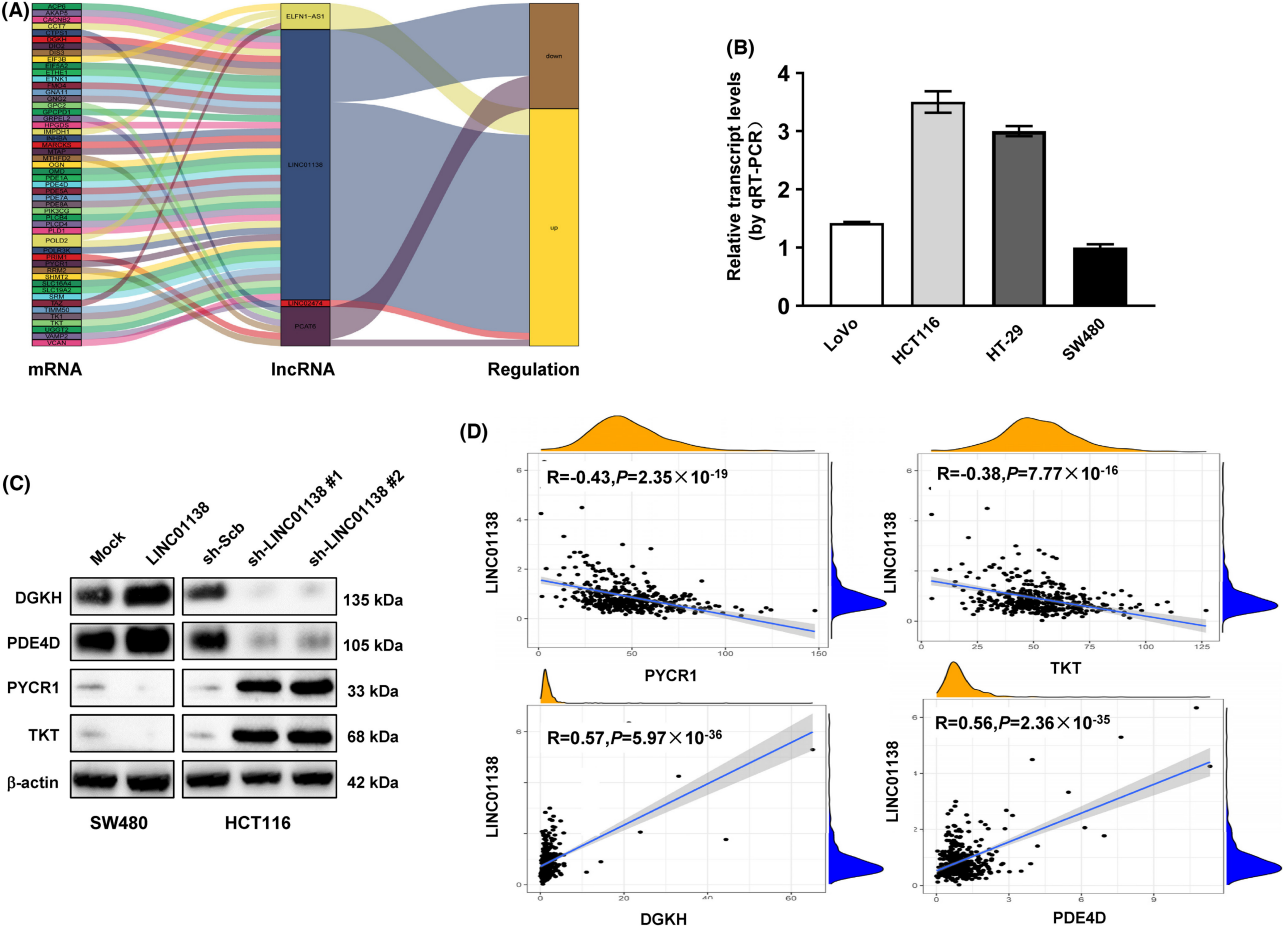
Correlation between LINC01138 and metabolism genes A, The alluvial diagram showing the correlation between metabolism related lncRNAs and metabolism genes. B, qRT‐PCR assays showing the levels of *LINC01138* in COAD cell lines (*n* = 3). C, Immunoblotting assays showing the levels of *EDGKH*, *PDE4D*, *PYCR1*, and *TK1* in COAD cells. D, The gene expression correlation between *LINC01138* and *DGKH*, *PDE4D, PYCR1*, and *TK1* in COAD. Pearson correlation analysis and statistics were done in GraphPad Prism 9 for Figure 9D.

We next investigated the links between these four lncRNAs and the metabolic genes (|coefficient| > 0.3). As shown in Figure 9A, *LINC01138* is the most closely lncRNA related to metabolic genes. Therefore, we initially selected *LINC01138* as a candidate gene to explore its role in COAD. First, based on endogenous expression level of *LINC01138* in cancer cell lines, we chose SW480 and HCT116 as the follow‐up research object (Figure 9B). As expected, enforced or impaired the expression of *LINC01138* affected metabolic genes expression in COAD cell lines (Figure 8C). The correlation between *LINC01138* and metabolic genes is shown in Figure 8D.

### 3.8 LINC01138 affects tumor progression in COAD in vitro

**FIGURE 10:**
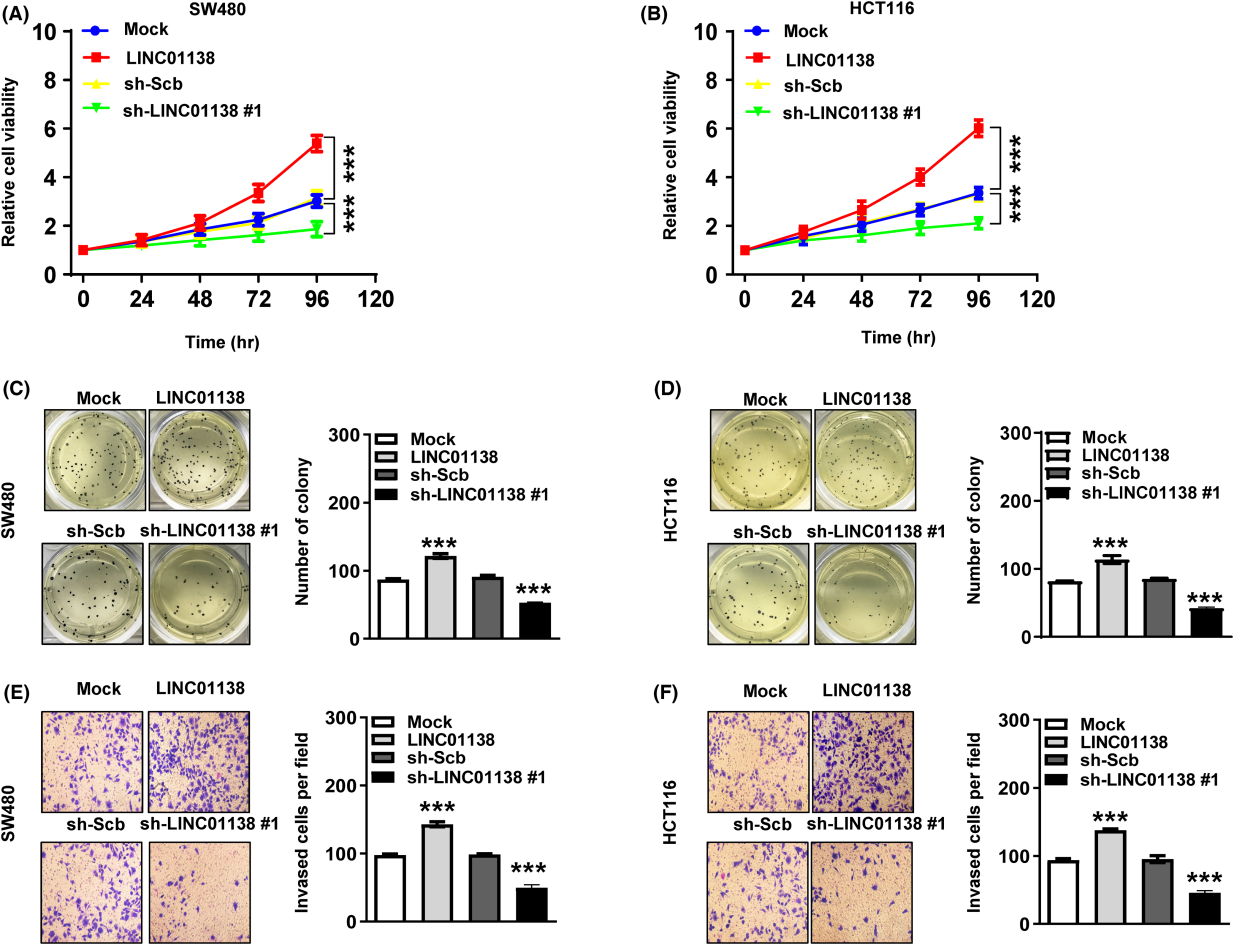
LINC01138 affects tumor progression in COAD cells. (A and B) MTT assay indicating the viability of SW480 and HCT116 cells treated stably transfected as indicated, respectively (*n* = 5). (C and D) Soft agar assays indicating the growth of SW480 and HCT116 cells treated with stably transfected as indicated, respectively (*n* = 3). (E and F) Matrigel invasion assays indicating the invasion of SW480 and HCT116 cells treated with stably transfected as indicated, respectively (*n* = 3). Unpaired two‐tailed *t*‐test was used to compare in Figure 10. ***p < 0.001.

To characterize the effects of *LINC01138* in COAD, cells with stable overexpression and knockdown of *LINC01138* were established. Overexpression of *LINC01138* accelerated cell viability of SW480. Moreover, a significantly increased in cell growth and invasion was observed in cultured SW480 cells following *LINC01138* overexpression (Figure 10A–F). Meanwhile, silencing the expression of *LINC01138* result in decreased in cell viability, growth, and invasion in HCT116 cells (Figure 10A–F). Taken together, these data showed that *LINC011381* affects tumor progression in COAD.

## 4 DISCUSSION

Cellular metabolic reprogramming is a common feature for cancer, allowing tumor cells to meet the demands of rapid proliferation or survival.^1^ For example, O‐GlcNAcylation activates PGK1 led to enhance glycolysis, and finally promote tumorigenesis in colon cancer.^24^ A new study demonstrated that UCP2 deficiency resulted in promoting phospholipid synthesis, which facilitates the transformation of malignant colon cells^.^ ^25^ These available studies show that target cancer metabolism maybe a promising way for cancer therapy.

LncRNA is a class of noncoding RNA with more than 200 nucleotides with no protein‐coding capacity.^12^ In recent years, aberranted lncRNAs have been found in various tumors including COAD, leading to the genesis and progression of cancer.^13^, ^14^, ^15^, ^16^, ^17^ However, the association between lncRNA and cancer cell metabolism in COAD is largely unknown. In this work, 152 differentially expressed lncRNAs were significantly associated with differentially expressed metabolism genes. Among them, 12 lncRNAs were associated with OS of COAD patients. Finally, based on the analyses of LASSO, a prognostic prediction model involving four metabolism‐related lncRNAs were established. Patients with COAD were randomly divided to training‐ and validation‐cohorts. According to the median risk score, the training cohort was divided to the high‐risk group and low‐risk group. Patients in the high‐risk group experienced worse OS than those in the low‐risk group. ROC curve analyses revealed that the accuracy of the MRlncRNAs signature. Univariate and multivariate analyses further suggested that the gene signature is an independent prognostic factor of COAD. Besides, in clinical subgroups, the lncRNAs signature also performed well. Finally, as a representative gene of *LINC01138*, our result showed that *LINC011381* affected tumor progression in COAD. In summary, this metabolic signature has a good ability to predict prognosis of COAD patients.

All four metabolism‐related lncRNAs (*ELFN1‐AS1*, *LINC01138*, *LINC02474*, and *PCAT6*) displayed a high expression in the high‐risk group. Among them, *ELFN1‐AS1*, *LINC02474*, and *PCAT6* have been reported to be associated with COAD.^26^, ^27^, ^28^ For example, *ELFN1‐AS1* acts as oncogene through sponging microRNAs in colorectal cancer.^26^ *LINC02474* by inhibiting GZMB expression to regulate progression of colorectal cancer.^27^ Since our study revealed that *LINC01138* was most closely with metabolism gene expression, we initially explored the function of *LINC01138*. Western blot assays revealed that forced *LINC01138* expression in colon cancer cells SW480 increased the expression of diacylglycerol kinase eta (*DGKH*) and Phosphodiesterase 4D (*PDE4D*), whereas resulted in decrease the expression of pyrroline‐5‐carboxylate reductase 1 (*PYCR1*) and transketolase (*TKT*). *LINC01138* knockdown in colon cancer cells HCT116 led to an opposite change in metabolic gene. This result is in line with correlation analysis from dataset. Aberranted *LINC01138* is found highly expression some tumors. A study showed that *LINC01138* by interacting with PRMT5 to facilitate tumor progression in hepatocellular carcinoma.^29^ Moreover, another report demonstrated that *LINC01138* affected cell growth in clear cell renal cell carcinoma through modulating lipid metabolism.^30^ However, the association between *LINC01138* and metabolism in COAD is unclarified. Here, we first characterized *LINC01138* is associated with the progression of COAD.

The four metabolism genes (*DGKH*, *PDE4D, PYCR1*, and *TKT*) are most closely associated with *LINC01138*. DGKH belongs to the diacylglycerol kinase enzyme family that participates in regulating the intracellular concentrations of diacylglycerol and phosphatidic acid.^31^ Roles of *DGKH* in COAD have not been fully discussed. However, Molatore et al. proved that *DGKH* was upregulated in rats with pheochromocytoma and may represent a potential biomarkers of the disease.^32^ Thus, *DGKH* may be important in COAD development. *PDE4D* is a subtype of metallohydrolases, which is involved in multiple tumor promoting processes^.^ ^33^ A previous study uncovered that miR‐139‐5p by decreasing the level of *PDE4D* led to suppress the growth of xenograft colorectal tumors.^34^ *PYCR1* is the key enzyme responsible for proline synthesis. Previous studies demonstrated that *PYCR1* is associated with the development of numerous cancers, including kidney cancer and hepatocellular carcinoma.^35^, ^36^ Yan et al. revealed that *PYCR1* is highly expressed in colorectal cancer, and reduced expression of *PYCR1* inhibits proliferation of colorectal cancer, suggest an oncogenic role of *PYCR1* in colorectal cancer.^37^ *TKT* is the key enzyme controlling the metabolic flux and direction of pentose phosphate pathway, which is required for maintaining cell proliferation in various cancers.^38^, ^39^ Meanwhile, a new study demonstrated that *TKT* promoted colorectal cancer cell growth and metastasis through modulating *AKT* phosphorylation.^40^ In the present study, our preliminary study suggested that *LINC01138* can regulate the level of metabolic genes, and combined with the role of *LINC01138* in COAD, we reasonably speculated that *LINC01138* may affect the progression of COAD by regulating metabolism.

Inevitably, our study had a few limitations. First, our work based on open accessed TCGA databases, and the prognostic model should be validated in additional independent samples. Second, the coexpression mechanisms of crucial lncRNA and metabolism genes should be clarified. Third, functional studies are required to elucidate our findings in this study.

## 5 CONCLUSIONS

In summary, we established a metabolism‐associated lncRNAs model to predict the prognosis in COAD patients. Our work may provide guidance for the treatment of COAD.

## AUTHOR CONTRIBUTIONS

**Yimin Sun:** Data curation (lead); funding acquisition (equal); methodology (lead); resources (lead); validation (lead); writing – original draft (equal); writing – review and editing (equal). **Bingyan Liu:** Data curation (lead); formal analysis (lead); investigation (lead); software (equal); validation (equal); writing – original draft (equal). **Baolai Xiao:** Formal analysis (equal); investigation (equal); methodology (equal); software (equal); validation (equal); visualization (equal); writing – original draft (equal). **Xuefeng Jiang:** Data curation (equal); formal analysis (equal); methodology (equal); writing – original draft (equal). **Jinjian Xiang:** Data curation (equal); formal analysis (equal); investigation (equal); software (equal); writing – original draft (equal). **Jianping Xie:** Formal analysis (equal); investigation (equal); supervision (equal); validation (equal); writing – original draft (equal). **Xiao Miao Hu:** Conceptualization (lead); funding acquisition (lead); methodology (equal); project administration (lead); writing – review and editing (lead).

## FUNDING INFORMATION

This work was supported by the Health and Family Planning Research Foundation of Hubei Province of China (grant no. WJ2016‐Y‐26).

## CONFLICT OF INTEREST

The authors declare no competing interests.

## Supporting information


Figure S1

Table S1.

Table S2.

## Data Availability

The datasets used and/or analysed in this study are available from the corresponding author on reasonable request.
