# Supplementary material for: Metabolism‐related lncRNAs signature to predict the prognosis of colon adenocarcinoma
Source: Cancer Med. 2022 Nov 10;12(5):5994–6008. doi: 10.1002/cam4.5412 (PMC10028123; doi:10.1002/cam4.5412)

Figure 1

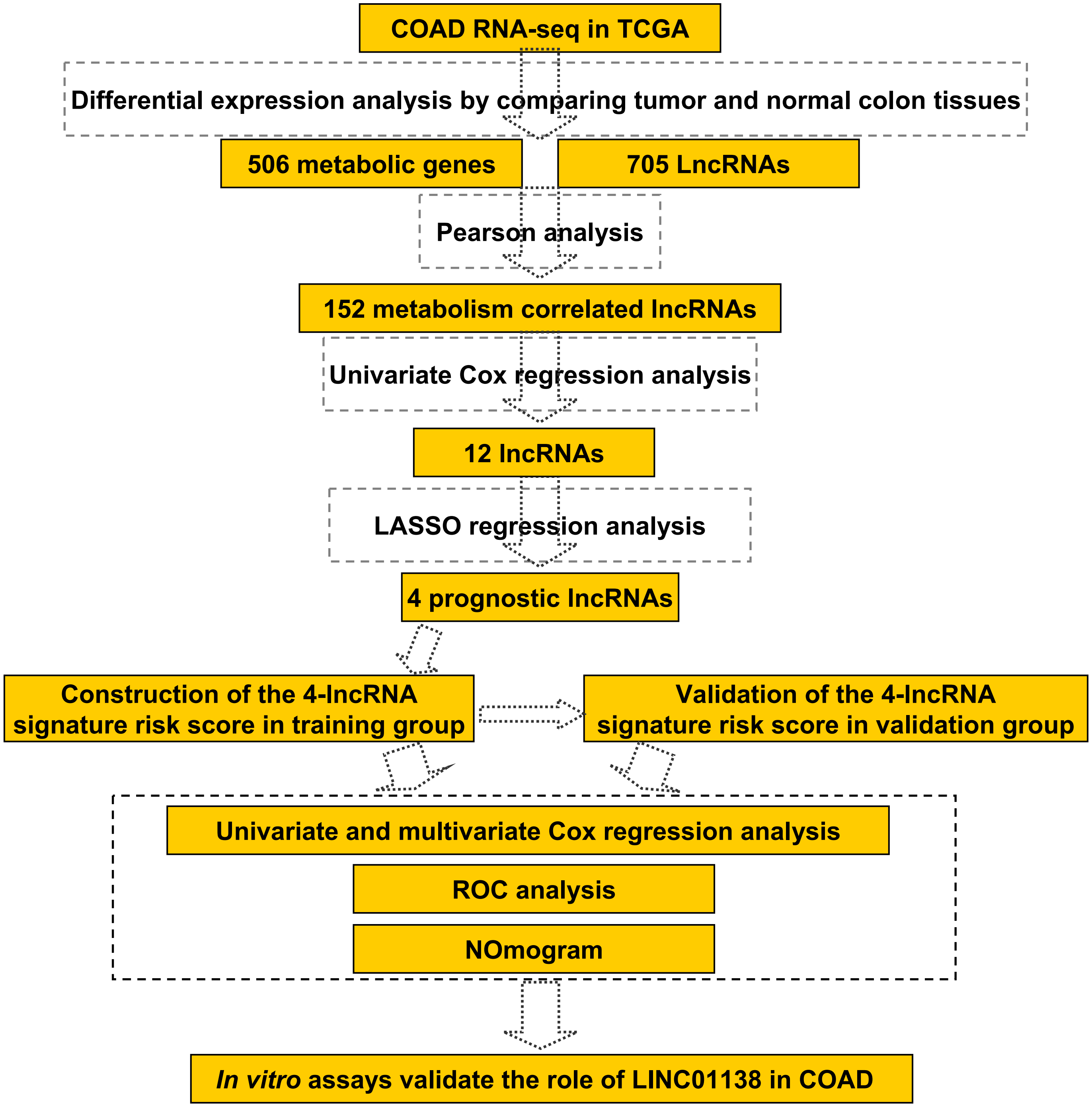

COAD RNA-seq in TCGA
Differential expression analysis by comparing tumor and normal colon tissues
506 metabolic genes
705 LncRNAs
Pearson analysis
152 metabolism correlated lncRNAs
Univariate Cox regression analysis
12 lncRNAs
LASSO regression analysis
4 prognostic lncRNAs
Construction of the 4-lncRNA signature risk score in training group
Validation of the 4-lncRNA signature risk score in validation group
Univariate and multivariate Cox regression analysis
ROC analysis
NOmogram
In vitro assays validate the role of LINC01138 in COAD

Figure 2

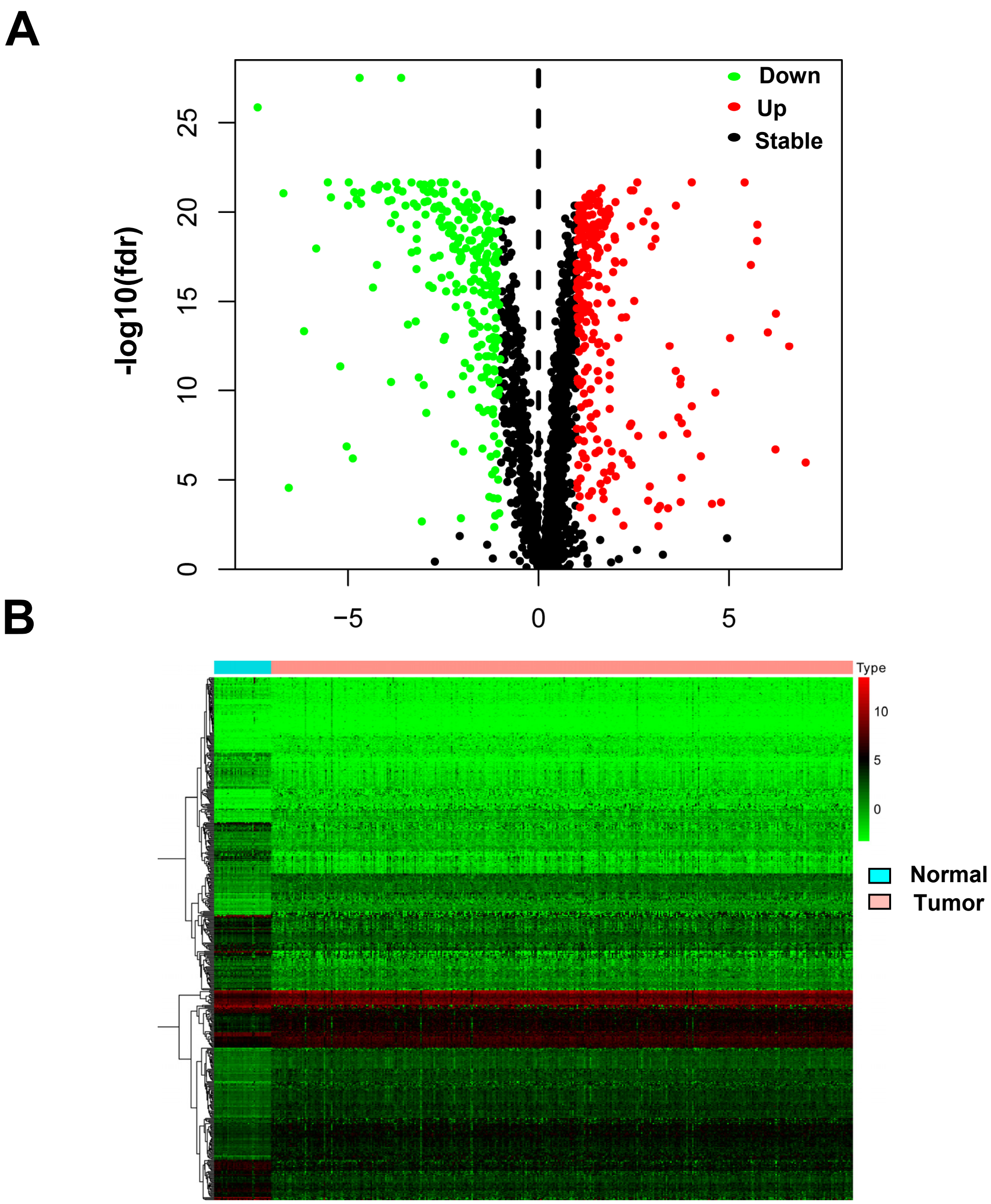
A
Down
Up
Stable
-log10(fdr)
0
5
10
15
20
25
−5
0
5
B
Type
10
5
0
Normal
Tumor

Figure 3

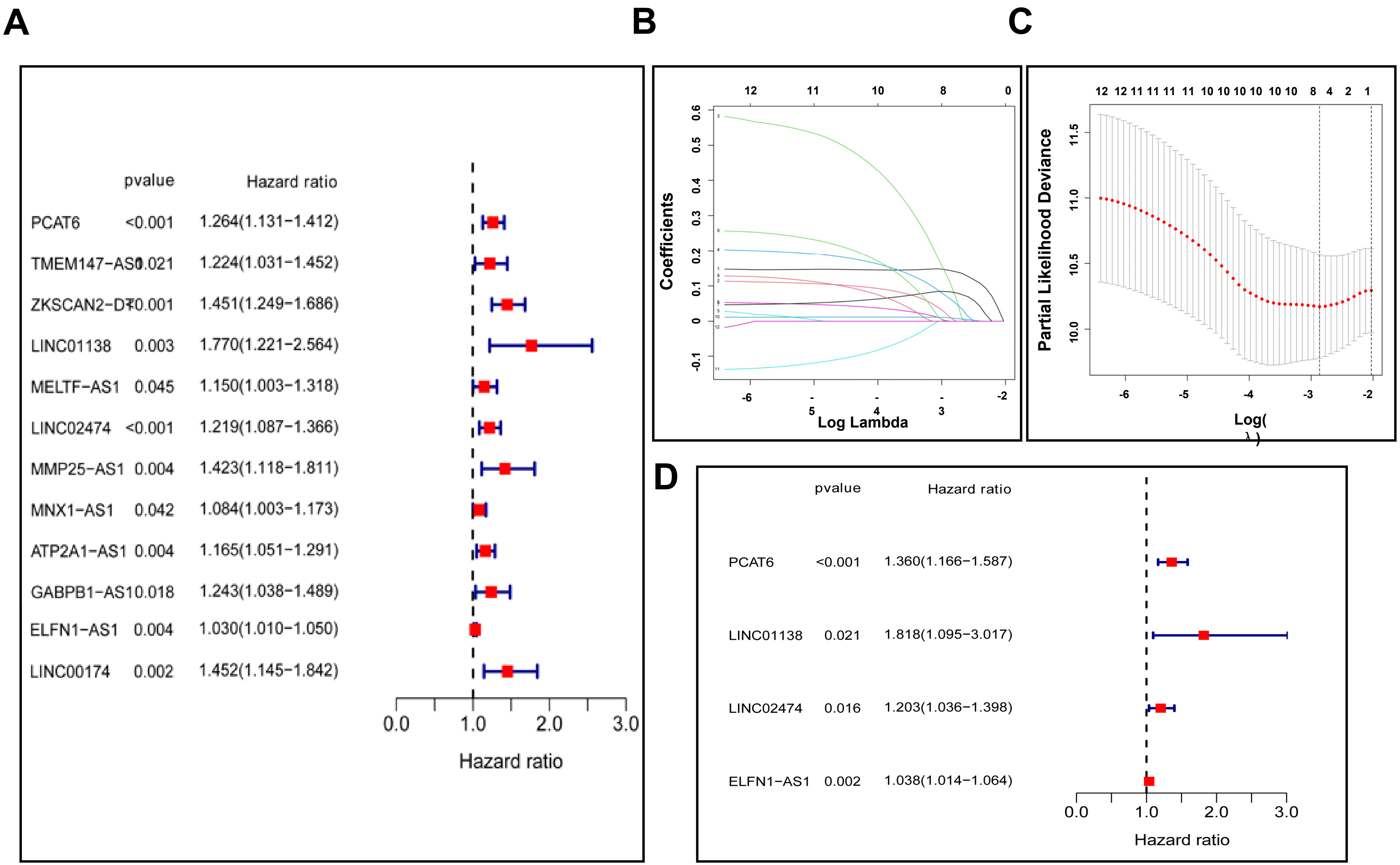

A
pvalue
Hazard ratio
PCAT6 <0.001 1.264(1.131−1.412)
TMEM147−AS1 0.021 1.224(1.031−1.452)
ZKSCAN2−DT <0.001 1.451(1.249−1.686)
LINC01138 0.003 1.770(1.221−2.564)
MELTF−AS1 0.045 1.150(1.003−1.318)
LINC02474 <0.001 1.219(1.087−1.366)
MMP25−AS1 0.004 1.423(1.118−1.811)
MNX1−AS1 0.042 1.084(1.003−1.173)
ATP2A1−AS1 0.004 1.165(1.051−1.291)
GABPB1−AS1 0.018 1.243(1.038−1.489)
ELFN1−AS1 0.004 1.030(1.010−1.050)
LINC00174 0.002 1.452(1.145−1.842)
0.0
1.0
2.0
3.0
Hazard ratio
B
12
11
10
8
0
Coefficients
Log Lambda
C
Partial Likelihood Deviance
Log(λ)
D
pvalue
Hazard ratio
PCAT6 <0.001 1.360(1.166−1.587)
LINC01138 0.021 1.818(1.095−3.017)
LINC02474 0.016 1.203(1.036−1.398)
ELFN1-AS1 0.002 1.038(1.014−1.064)
0.0
1.0
2.0
3.0
Hazard ratio

Figure 4

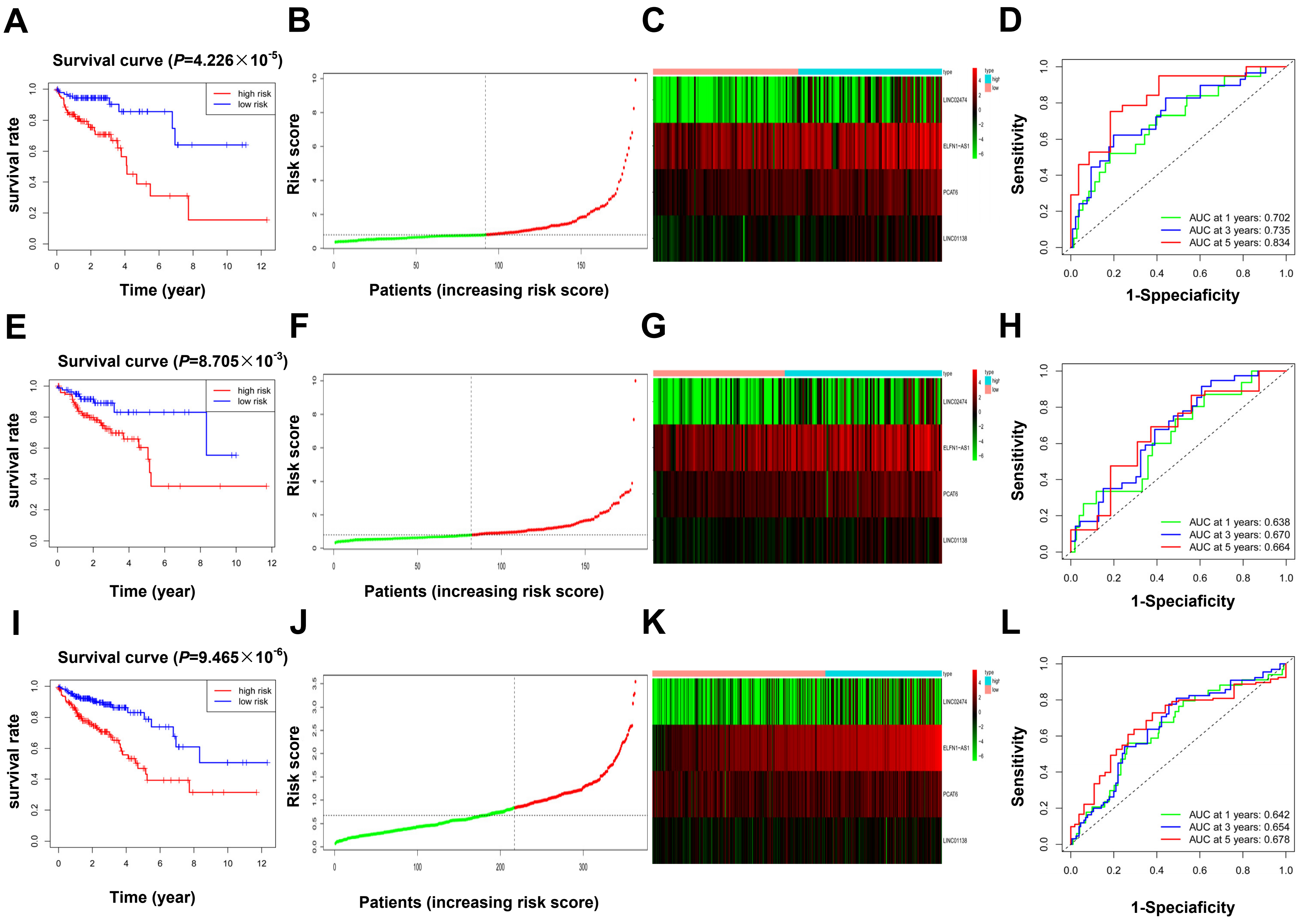

A
B
C
D
Survival curve (P=4.226×10^{-5})
high risk
low risk
survival rate
Time (year)
Risk score
Patients (increasing risk score)
type
high
low
LINC02474
ELFN1−AS1
PCAT6
LINC01138
Sensitivity
1-Sppeciaficity
AUC at 1 years: 0.702
AUC at 3 years: 0.735
AUC at 5 years: 0.834
E
F
G
H
Survival curve (P=8.705×10^{-3})
high risk
low risk
survival rate
Time (year)
Risk score
Patients (increasing risk score)
Sensitivity
1-Speciaficity
AUC at 1 years: 0.638
AUC at 3 years: 0.670
AUC at 5 years: 0.664
I
J
K
L
Survival curve (P=9.465×10^{-6})
high risk
low risk
survival rate
Time (year)
Risk score
Patients (increasing risk score)
Sensitivity
1-Speciaficity
AUC at 1 years: 0.642
AUC at 3 years: 0.654
AUC at 5 years: 0.678

Figure 5

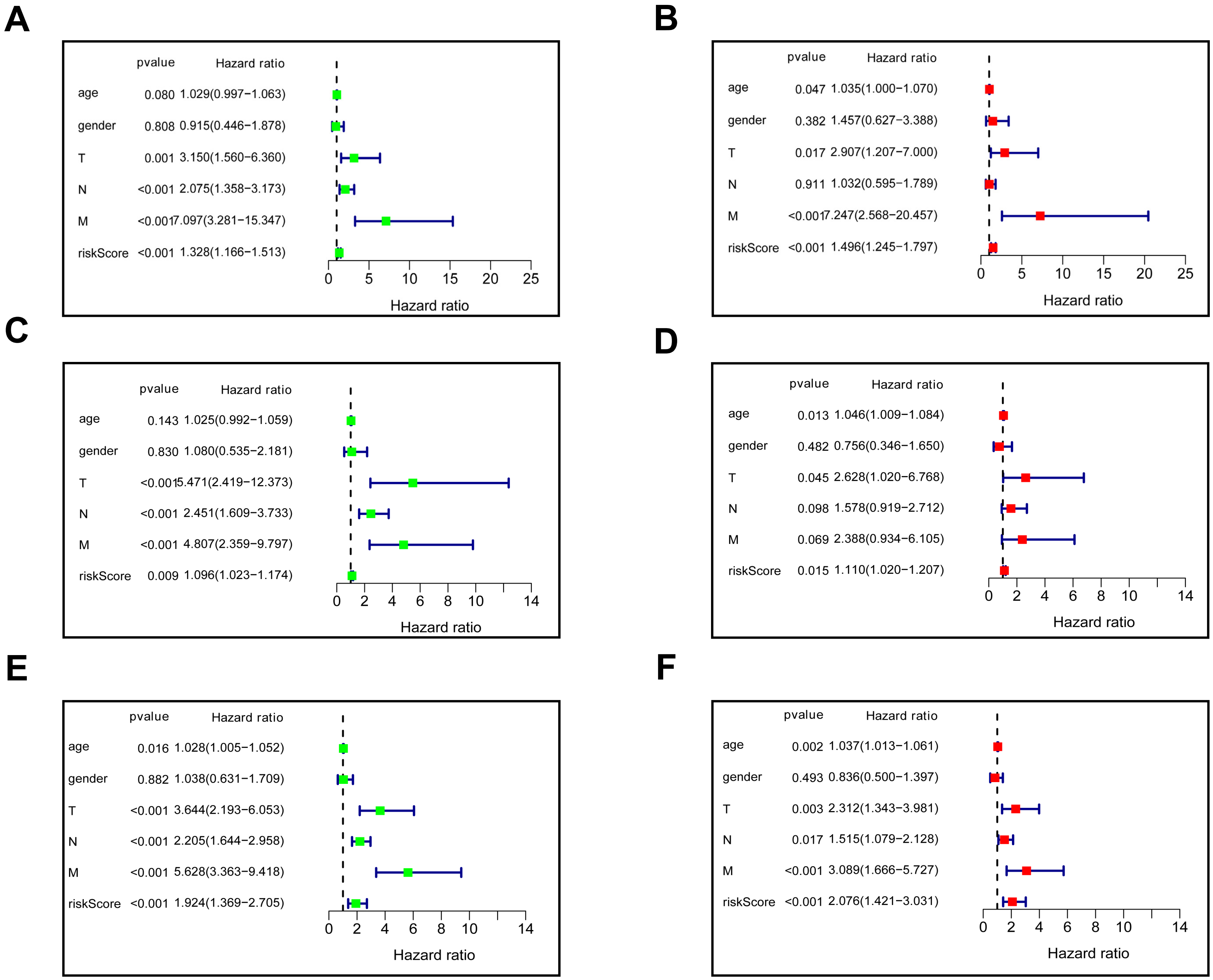

A
pvalue Hazard ratio
age 0.080 1.029(0.997−1.063)
gender 0.808 0.915(0.446−1.878)
T 0.001 3.150(1.560−6.360)
N <0.001 2.075(1.358−3.173)
M <0.0017.097(3.281−15.347)
riskScore <0.001 1.328(1.166−1.513)
0 5 10 15 20 25
Hazard ratio
B
pvalue Hazard ratio
age 0.047 1.035(1.000−1.070)
gender 0.382 1.457(0.627−3.388)
T 0.017 2.907(1.207−7.000)
N 0.911 1.032(0.595−1.789)
M <0.0017.247(2.568−20.457)
riskScore <0.001 1.496(1.245−1.797)
0 5 10 15 20 25
Hazard ratio
C
pvalue Hazard ratio
age 0.143 1.025(0.992−1.059)
gender 0.830 1.080(0.535−2.181)
T <0.0015.471(2.419−12.373)
N <0.001 2.451(1.609−3.733)
M <0.001 4.807(2.359−9.797)
riskScore 0.009 1.096(1.023−1.174)
0 2 4 6 8 10 14
Hazard ratio
D
pvalue Hazard ratio
age 0.013 1.046(1.009−1.084)
gender 0.482 0.756(0.346−1.650)
T 0.045 2.628(1.020−6.768)
N 0.098 1.578(0.919−2.712)
M 0.069 2.388(0.934−6.105)
riskScore 0.015 1.110(1.020−1.207)
0 2 4 6 8 10 14
Hazard ratio
E
pvalue Hazard ratio
age 0.016 1.028(1.005−1.052)
gender 0.882 1.038(0.631−1.709)
T <0.001 3.644(2.193−6.053)
N <0.001 2.205(1.644−2.958)
M <0.001 5.628(3.363−9.418)
riskScore <0.001 1.924(1.369−2.705)
0 2 4 6 8 10 14
Hazard ratio
F
pvalue Hazard ratio
age 0.002 1.037(1.013−1.061)
gender 0.493 0.836(0.500−1.397)
T 0.003 2.312(1.343−3.981)
N 0.017 1.515(1.079−2.128)
M <0.001 3.089(1.666−5.727)
riskScore <0.001 2.076(1.421−3.031)
0 2 4 6 8 10 14
Hazard ratio

Figure 6

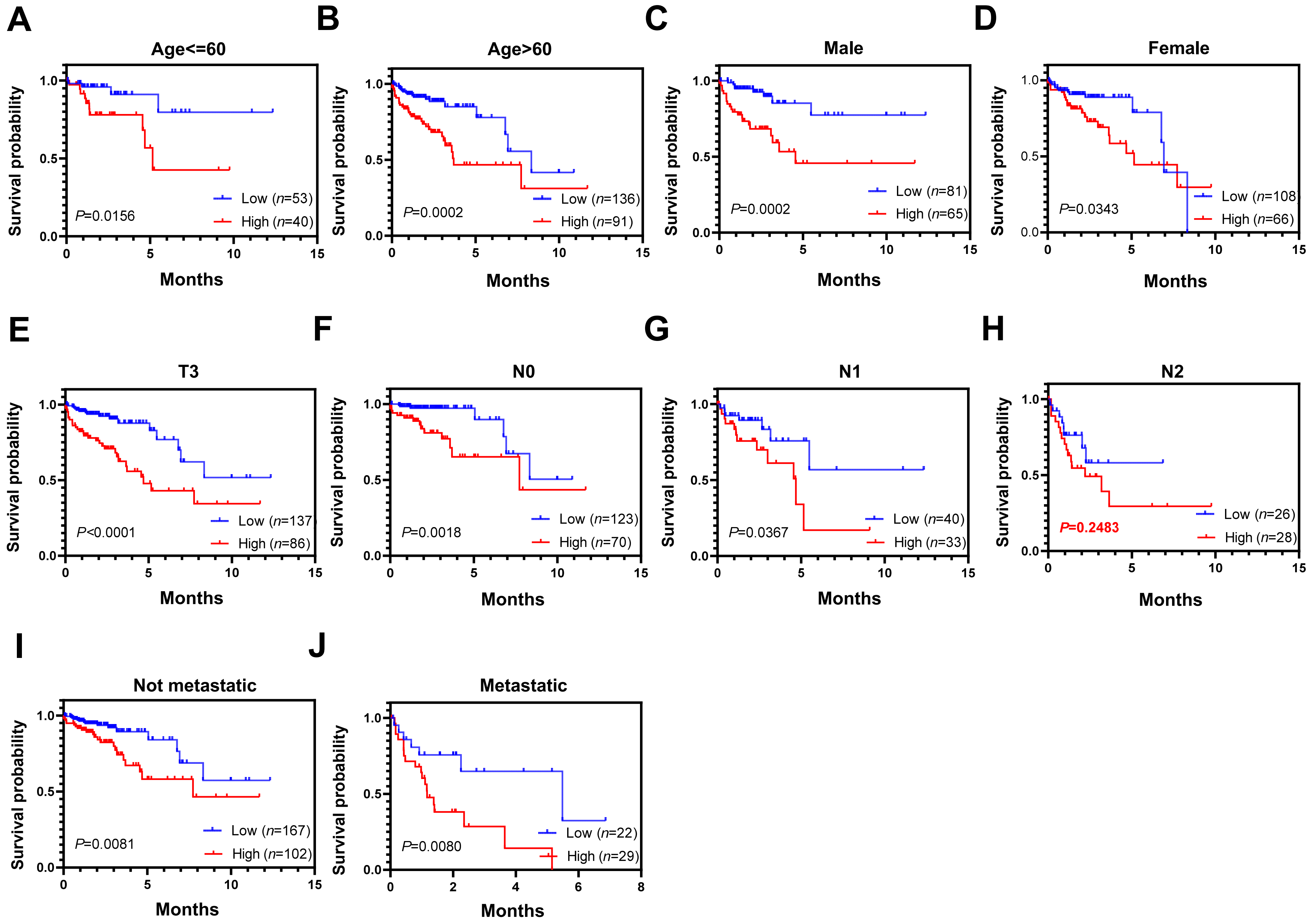
A
Age<=60
Survival probability
Months
P=0.0156
Low (n=53)
High (n=40)
B
Age>60
P=0.0002
Low (n=136)
High (n=91)
C
Male
P=0.0002
Low (n=81)
High (n=65)
D
Female
P=0.0343
Low (n=108)
High (n=66)
E
T3
P<0.0001
Low (n=137)
High (n=86)
F
N0
P=0.0018
Low (n=123)
High (n=70)
G
N1
P=0.0367
Low (n=40)
High (n=33)
H
N2
P=0.2483
Low (n=26)
High (n=28)
I
Not metastatic
P=0.0081
Low (n=167)
High (n=102)
J
Metastatic
P=0.0080
Low (n=22)
High (n=29)

Figure 7

A

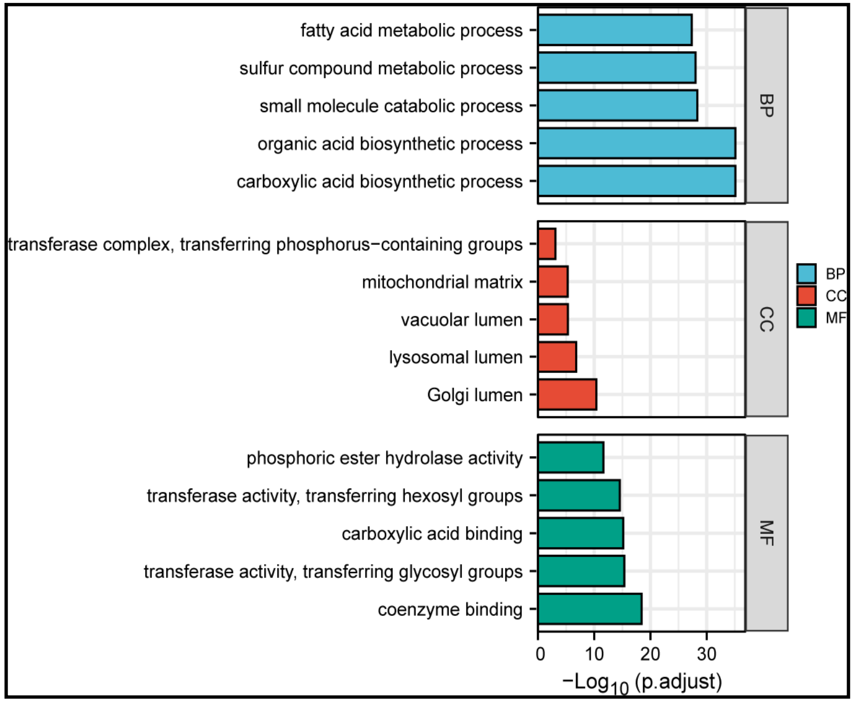
fatty acid metabolic process
sulfur compound metabolic process
small molecule catabolic process
organic acid biosynthetic process
carboxylic acid biosynthetic process
transferase complex, transferring phosphorus−containing groups
mitochondrial matrix
vacuolar lumen
lysosomal lumen
Golgi lumen
phosphoric ester hydrolase activity
transferase activity, transferring hexosyl groups
carboxylic acid binding
transferase activity, transferring glycosyl groups
coenzyme binding
BP
CC
MF
0
10
20
30
−Log10 (p.adjust)

B

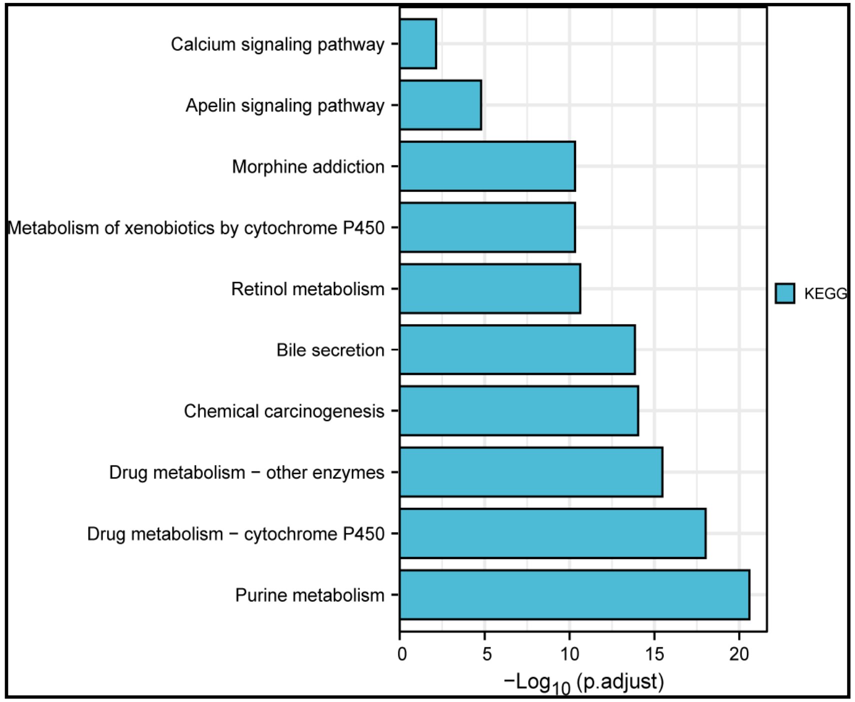
Calcium signaling pathway
Apelin signaling pathway
Morphine addiction
Metabolism of xenobiotics by cytochrome P450
Retinol metabolism
Bile secretion
Chemical carcinogenesis
Drug metabolism − other enzymes
Drug metabolism − cytochrome P450
Purine metabolism
KEGG
0
5
10
15
20
−Log10 (p.adjust)

Figure 8

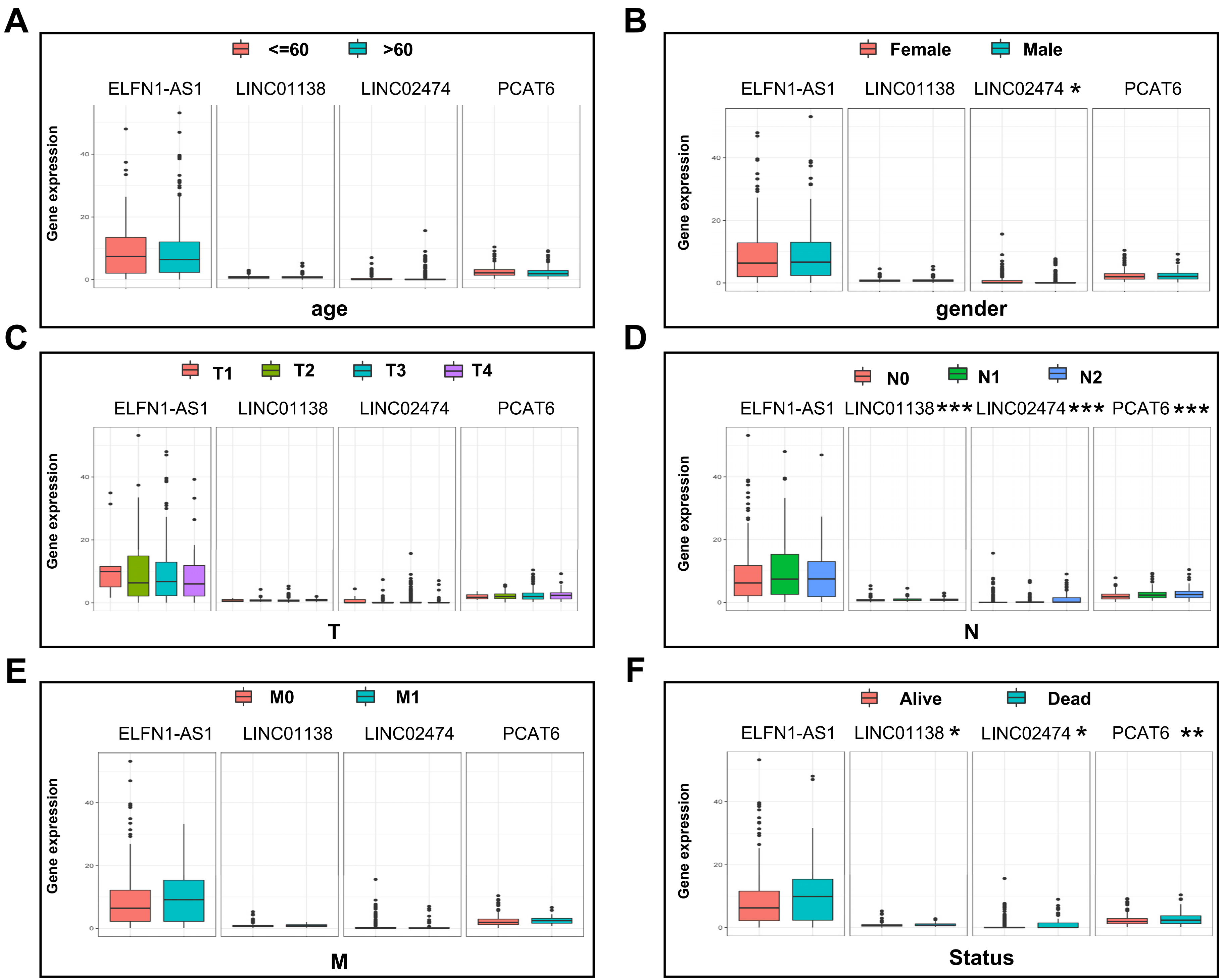
A
<=60
>60
ELFN1-AS1
LINC01138
LINC02474
PCAT6
Gene expression
age
B
Female
Male
ELFN1-AS1
LINC01138
LINC02474 *
PCAT6
Gene expression
gender
C
T1
T2
T3
T4
ELFN1-AS1
LINC01138
LINC02474
PCAT6
Gene expression
T
D
N0
N1
N2
ELFN1-AS1
LINC01138 ***
LINC02474 ***
PCAT6 ***
Gene expression
N
E
M0
M1
ELFN1-AS1
LINC01138
LINC02474
PCAT6
Gene expression
M
F
Alive
Dead
ELFN1-AS1
LINC01138 *
LINC02474 *
PCAT6 **
Gene expression
Status

Figure 9

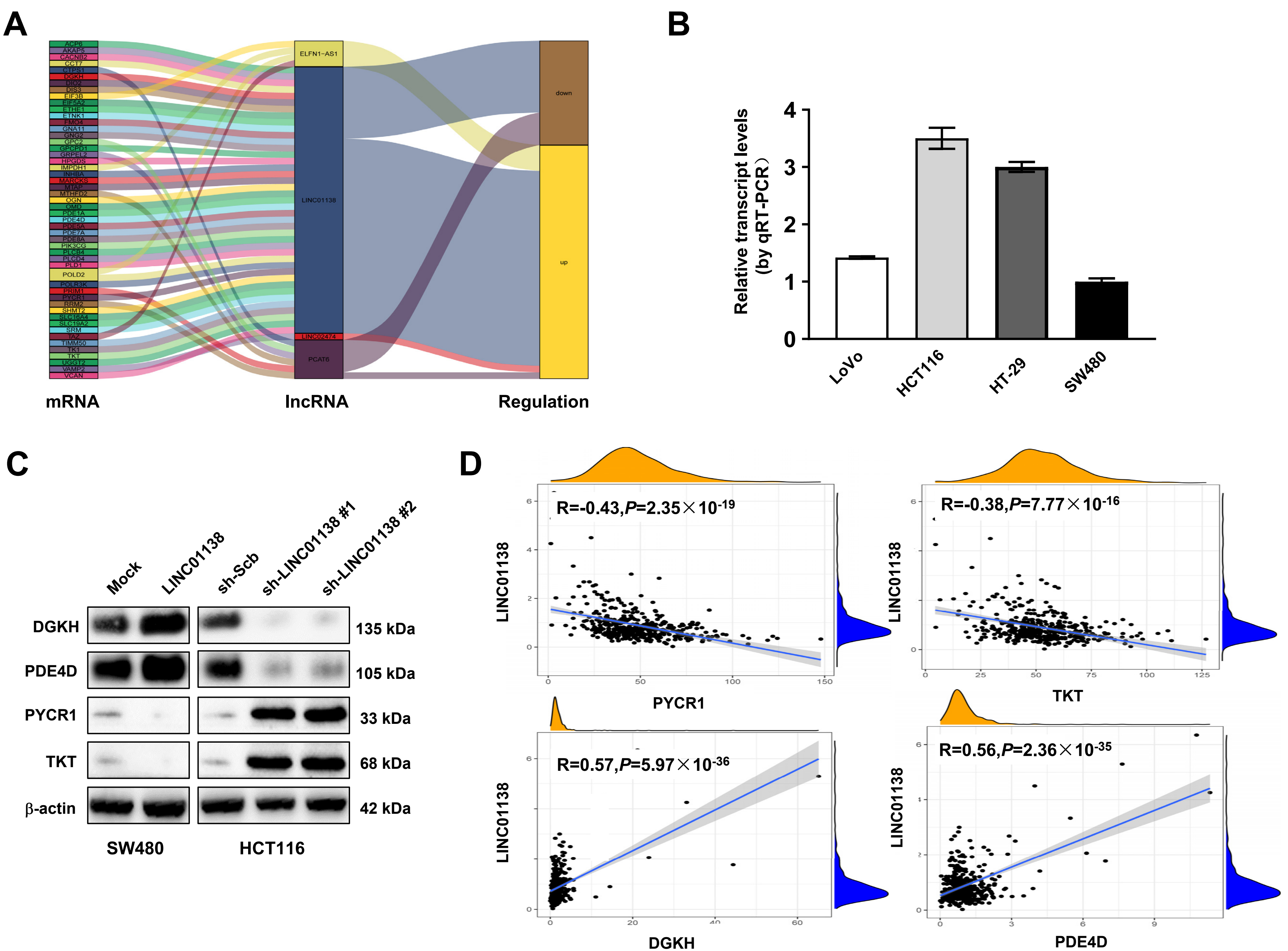

A
mRNA
lncRNA
Regulation
ELFN1-AS1
LINC01138
LINC02474
PCAT6
down
up
B
Relative transcript levels (by qRT-PCR)
LoVo
HCT116
HT-29
SW480
C
Mock
LINC01138
sh-Scb
sh-LINC01138 #1
sh-LINC01138 #2
DGKH
PDE4D
PYCR1
TKT
β-actin
135 kDa
105 kDa
33 kDa
68 kDa
42 kDa
SW480
HCT116
D
R=-0.43,P=2.35×10-19
R=-0.38,P=7.77×10-16
R=0.57,P=5.97×10-36
R=0.56,P=2.36×10-35
LINC01138
PYCR1
TKT
DGKH
PDE4D

Figure 10

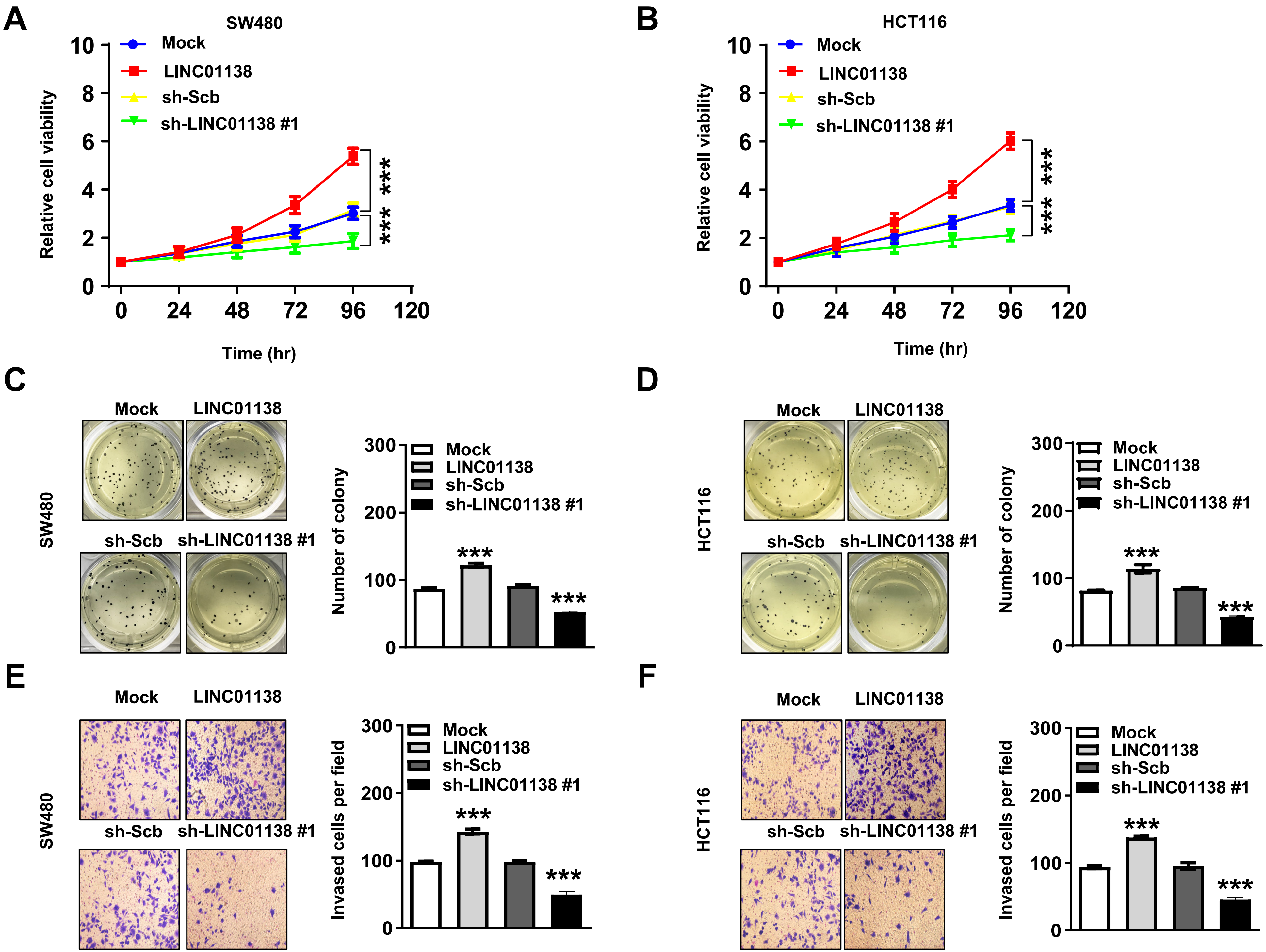
A
SW480
Mock
LINC01138
sh-Scb
sh-LINC01138 #1
Relative cell viability
Time (hr)
***
***
B
HCT116
Mock
LINC01138
sh-Scb
sh-LINC01138 #1
Relative cell viability
Time (hr)
***
***
C
SW480
Mock
LINC01138
sh-Scb
sh-LINC01138 #1
Number of colony
***
***
D
HCT116
Mock
LINC01138
sh-Scb
sh-LINC01138 #1
Number of colony
***
***
E
SW480
Mock
LINC01138
sh-Scb
sh-LINC01138 #1
Invased cells per field
***
***
F
HCT116
Mock
LINC01138
sh-Scb
sh-LINC01138 #1
Invased cells per field
***
***

Supplementary Figure

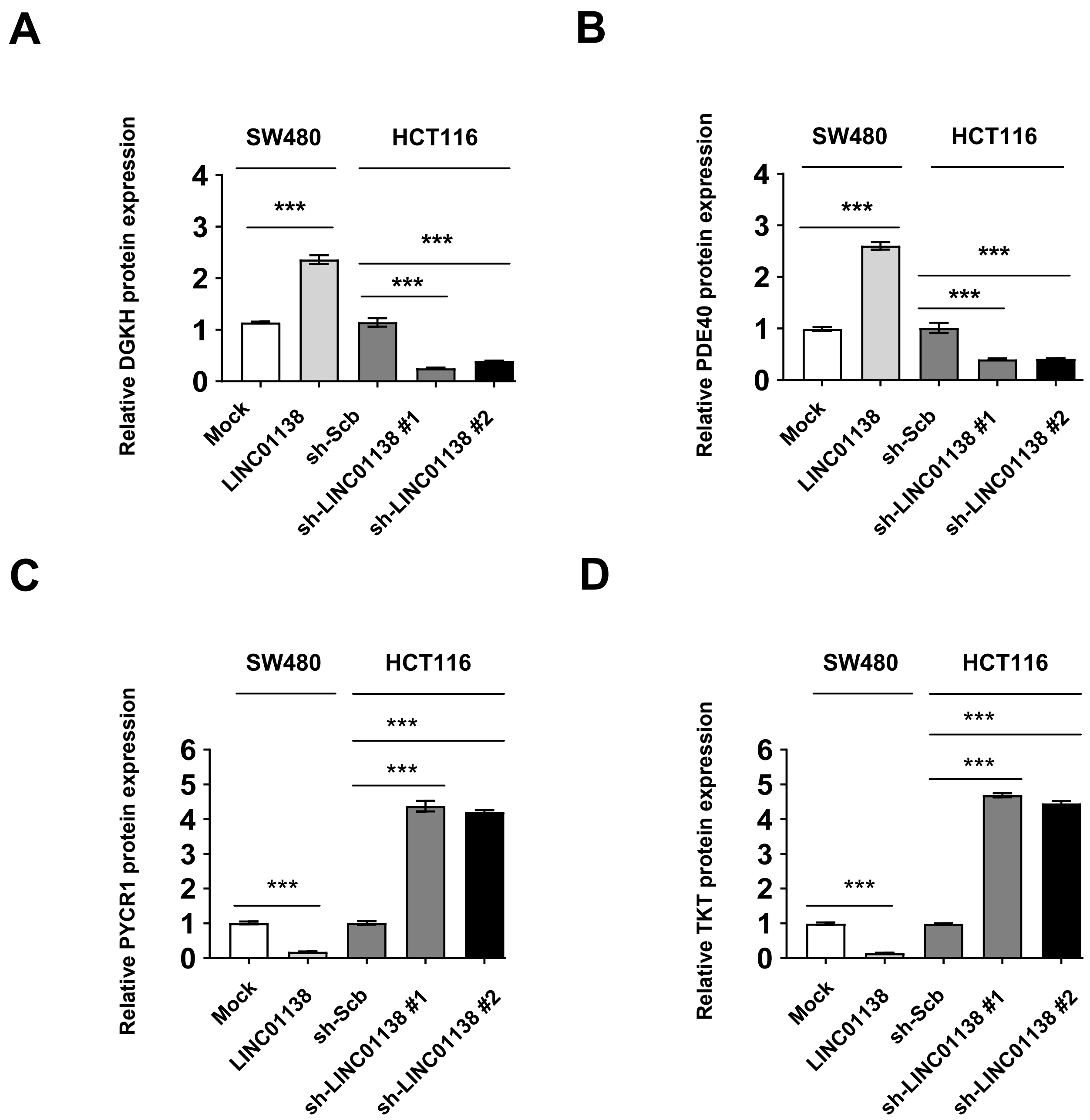

Supplement: Supplementary file 1 — Figure S1 [file CAM4-12-5994-s003.pdf]
